# Investigating the structure–activity relationship of marine natural polyketides as promising SARS-CoV-2 main protease inhibitors†

**DOI:** 10.1039/d1ra05817g

**Published:** 2021-09-22

**Authors:** Amr El-Demerdash, Ahmed A. Al-Karmalawy, Tarek Mohamed Abdel-Aziz, Sameh S. Elhady, Khaled M. Darwish, Ahmed H. E. Hassan

**Affiliations:** Chemistry Department, Organic Chemistry Division, Faculty of Science, Mansoura University Mansoura 35516 Egypt eldemerdash555@gmail.com; Metabolic Biology & Biological Chemistry Department, John Innes Centre Norwich Research Park Norwich NR4 7UH UK; Department of Pharmaceutical Medicinal Chemistry, Faculty of Pharmacy, Horus University-Egypt New Damietta 34518 Egypt akarmalawy@horus.edu.eg; Department of Cellular and Integrative Physiology, University of Texas Health Science Centre at San Antonio San Antonio Texas 78229-3900 USA; Zoology Department, Faculty of Science, Minia University El-Minia 61519 Egypt; Department of Natural Products, Faculty of Pharmacy, King Abdulaziz University Jeddah Saudi Arabia; Department of Medicinal Chemistry, Faculty of Pharmacy, Suez Canal University Ismailia Egypt; Department of Medicinal Chemistry, Faculty of Pharmacy, Mansoura University Mansoura 35516 Egypt ahmed_hassan@mans.edu.eg

## Abstract

Since its first report in December 2019, the novel coronavirus virus, SARS-CoV-2, has caused an unprecedented global health crisis and economic loss imposing a tremendous burden on the worldwide finance, healthcare system, and even daily life. Even with the introduction of different preventive vaccines, there is still a dire need for effective antiviral therapeutics. Nature has been considered as the historical trove of drug discovery and development, particularly in cases of worldwide crises. Herein, a comprehensive *in silico* investigation of a highly focused chemical library of 34 pederin-structurally related marine compounds, belonging to four polyketides families, was initiated against the SARS-CoV-2 main protease, Mpro, being the key replicating element of the virus and main target in many drugs development programs. Two of the most potent SARS-CoV-2 Mpro co-crystallized inhibitors, O6K and N3, were added to the tested database as reference standards. Through molecular docking simulation, promising compounds including Pederin (1), Dihydro-onnamide A (11), Onnamide C (14), Pseudo-onnamide A (17), and Theopederin G (29) have been identified from different families based on their superior ligand–protein energies and relevant binding profiles with the key Mpro pocket residues. Thermodynamic behaviors of the identified compounds were investigated through 200 ns all-atom molecular dynamics simulation illustrating their significant stability and pocket accommodation. Furthermore, structural activity preferentiality was identified for the pederin-based marine compounds highlighting the importance of the terminal guanidine and cyclic hemiacetal linker, and the length of the sidechain. Our findings highlight the challenges of targeting SARS-CoV-2 Mpro as well as recommending further *in vitro* and *in vivo* studies regarding the examined marine products either alone or in combination paving the way for promising lead molecules.

## Abstract 2

Marine natural polyketides showed promising SARS-CoV-2 main protease inhibitory activities.

## Graphical abstract

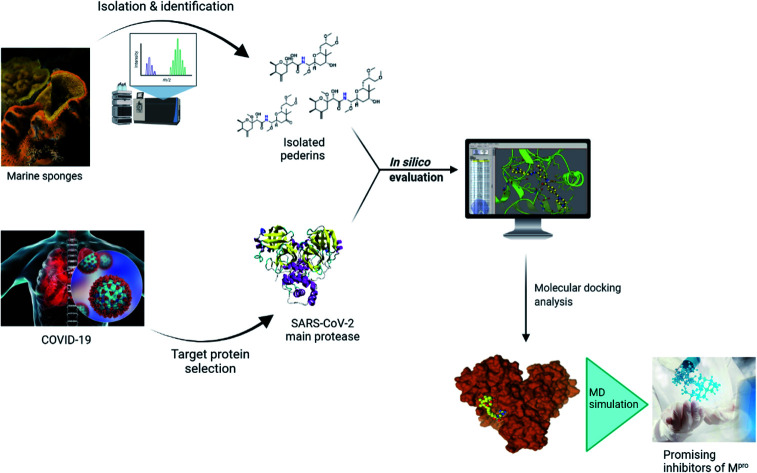

## 1. Introduction

The coronavirus disease 2019 (COVID-19) outbreak declared as a pandemic by World Health Organisation (WHO) in March 2020 is caused by the Severe Acute Respiratory Syndrome Coronavirus 2 (SARS-CoV-2), a newly identified coronavirus, which was first recognized in Wuhan, China. The global pandemic of SARS-CoV-2 is still ongoing. Although effective vaccines and vaccination programs are underway, there is an urgent need to identify new effective therapeutic agents to treat SARS-CoV-2 infection. SARS-CoV-2 is the seventh CoV capable of infecting humans (HCoV), but the first and only HCoV with pandemic potential.^1^ The previous six human pathogenic HCoVs are HCoV-229E, HCoV-OC43, severe acute respiratory syndrome coronavirus (SARS-CoV), HCoV-HKU1, HCoV-NL63, and Middle East respiratory syndrome (MERS-CoV).^1,2^ SARS-CoV-2 is a 100 nanometer in diameter pleomorphic circular-shaped protein complex that contains approximately 100 units of 10 nanometer length trimeric spike proteins on each virion.^3,4^ The structure SARS-CoV-2 consists of different structural and non-structural proteins to include spike (S) glycoprotein, envelop (E), membrane (M), nucleocapsid (N), hemagglutinin esterase dimer (HE), and non-structural proteins (NSP).^5^

The SARS-CoV-2 main protease (Mpro) is a non-structural protein that proteolytically cuts the overlapping pp1a and pp1ab polyproteins resulting in functional proteins that are required to mediate viral replication and transcription.^6^ Since the Mpro of SARS-CoV-2 is a very important key for the conversion of its primary polypeptides into essential functional proteins through its master role in both viral transcription and replication processes.^7,8^ Therefore, targeting SARS-CoV-2 Mpro is a very promising approach to get a lead compound combating the COVID-19 pandemic as soon as possible.^9–11^

Considering that oceans and seas are covering almost 70% of the planet's surface, the marine environment had privileged to represent the extremely largest ecological system on the earth with 92% of total phyla exists in life, is featuring a huge number of wealthy and advantageous produces yet undiscovered.^12–16^ Over seven decades and since the emerging of the first marine bioactive nucleotides in the 1950s by Brigman *et al.*, marine natural products (MNPs) have been marked as sustainable prolific pipelines for numerous structurally diverse bioactive candidates. Since more than 35 000 discovered compounds bearing unique structural features and unprecedented biological potentialities along with hundreds of new chemical entities are being unearthed each year, MNPs have preliviged a central focus of the worldwide drug lead discovery program.^17–20^ Those exploitations have led to the identification of 15 successful approved/commercialized marine-based drug leads including, along with more than 33 candidates that are currently under different preclinical investigations.^21–23^

Particularly, in 2020, the FAD has approved two marine-based compounds as anticancer, including belantamab mafodotin-blmf (Blenrep™), a synthetic drug-conjugate analog of the marine peptide dolastatin 10, originally isolated from a mollusk-derived cyanobacterium, recently developed by GlaxoSmithKline (GSK) and approved for the treatment of relapsed or refractory multiple myeloma.^24^ Lurbinectedin, a synthetic tetrahydropyrrolo [4,3,2-*de*]quinolin-8(1*H*)-one analog of the marine alkaloid trabectedin, which was originally isolated from the tunicate *Ecteinascidia turbinate*, recently defined by PharmaMar and approved for the treatment of metastatic small cell lung carcinoma.^25^

**Scheme 1:**
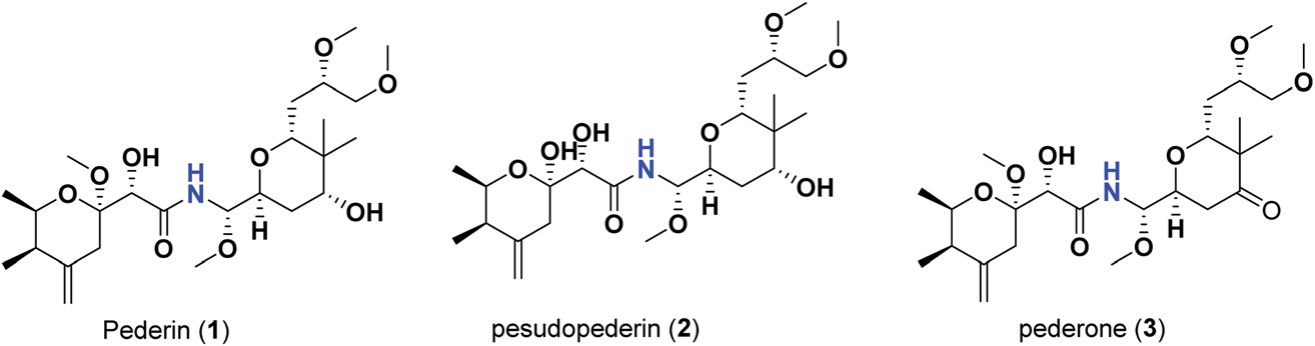
Isolated pederins (1–3).

**Scheme 2:**
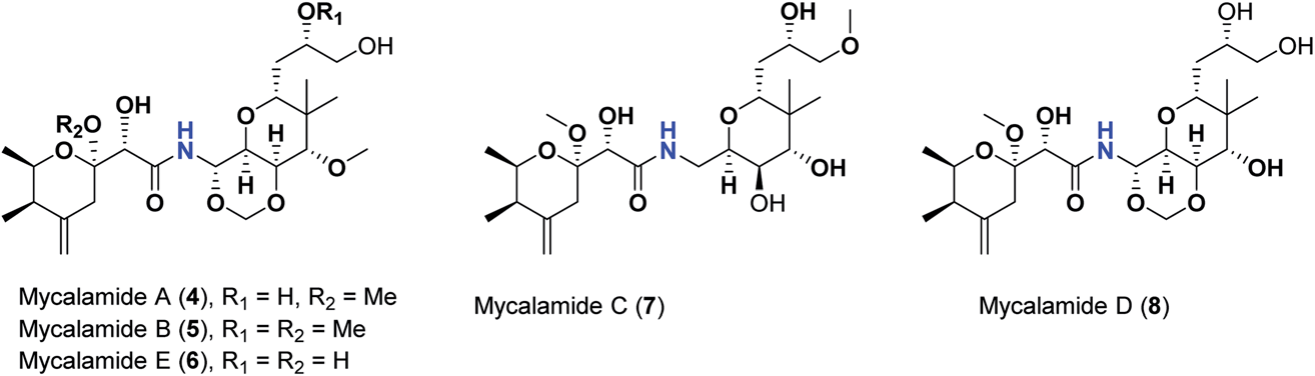
Isolated mycalamides (4–8).

**Scheme 3:**
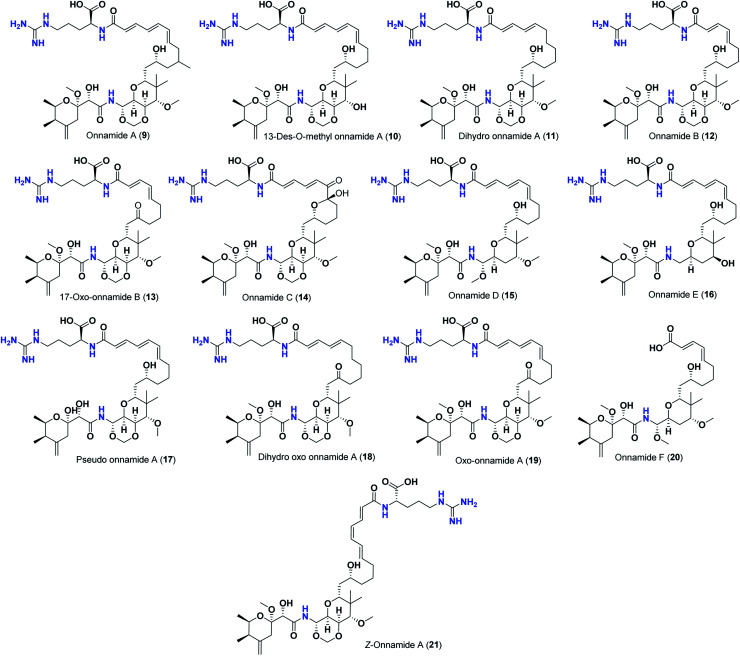
Isolated onnamides (9–21).

**Scheme 4:**
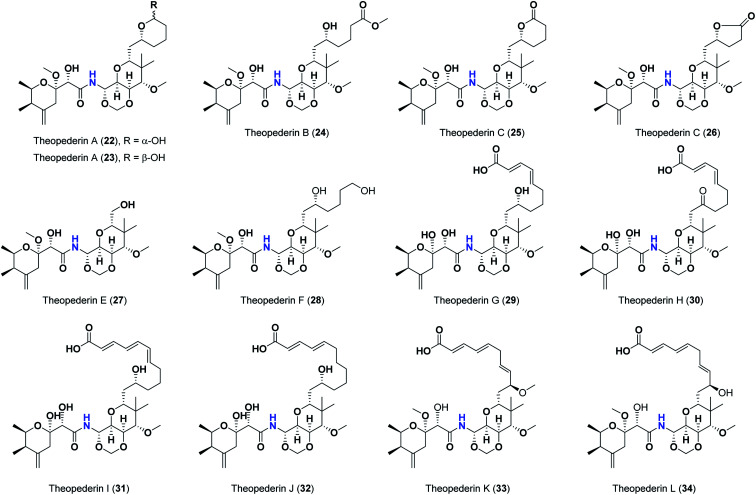
Reported theopederins (22–33).

Pederins/mycalamides/onnamides and theopederins are distinct families of structurally related polyketides-containing nitrogen which are biosynthetically derived from a PKS-NRPS gene cluster (Schemes 1–4). Chemically, they compromise the main core of two tetrahydropyran rings linked together through an *N*-acyl aminal and decorated by variant oxidation degrees.^26^ Pederin (1) was the first member to be reported in 1949 from the female beetle called *Paederus littoralis*, however, its structure was complete and revised in 1968.^27^ Later, in 1989–1990, perry *et al.* reported the isolation of structurally related members of marine origin, named mycalamides A–B (4–5) from a marine sponge of the genus *Mycale* collected in New Zealand.^28,29^ Subsequentially, two additional compounds, mycalamide C–D (7–8) were isolated from marine sponges of the genus *Stylinos* and *Mycale* sp.^30,31^ Venturi *et al.*, isolated the last member of this family namely mycalamide E (6) from the marine sponge *Mycale hentscheli* collected in Pelorus Sound, New Zealand.^32^ Onnamides (9–21) are considered the largest group of this family. They have been isolated from the marine sponges of the genus *Theonella* and *Trachycladus*.^33–36^ Theopederins A–J (22–31), were reported from marine sponges of the genus *Theonella*,^37,38^ however, theopederins K–L (32–33) were recorded from marine sponge *Discodermia* sp.^39^

Pharmacologically, pederin-related compounds are classified as protein synthesis inhibitors,^32,40,41^ which preliviged them to display a myriad of biological activities including cytotoxicity with IC_50_ under 5 nM (ref. 42–44) and immunosuppressive.^45^ Pederin and related derivatives were tested on a panel of tumor mammalian cells including HeLa and KB and showed significant cytotoxicity at 2 nM concentrations.^46^ Recently, Nakabachi *et al.* reported the potent cytotoxic activity of diaphorin, a structurally related pederin derivative isolated from a bacterial symbiont of the Asian citrus psyllid against 39 human cancer cells with GI_50_, TGI, and LC_50_ values at the micromolar range.^47^ More interestingly, they display *in vitro* potent antiviral activities. Mycalamide A (4), exhibited promising antiviral activity against A59 coronavirus. Moreover, mycalamides A–B (4–5) exhibited significant antiviral inhibition activity against Herpes simplex type-1 and Polio type-1 viruses active at 3.5–5.0 and 1.0–2.0 ng per disk respectively.^28,29^ Burres *et al.*, have demonstrated that these compounds can inhibit DNA and RNA replication, meanwhile, further reports documented their action to be more like protein synthesis inhibitors rather than DNA or RNA synthesis inhibitors.^40^ Additional four synthetic analogs of mycalamide A (4) were evaluated as strong protein synthesis inhibitors and showed potent binding to the nucleoprotein of the influenza virus (H1N1) and prevent its replication.^48^ For detailed chemistry including the isolation, synthesis, and biological potentialities of those families of natural products, see the comprehensive report by Mosey *et al.*^26^

Therefore, taking into consideration the crucial role of SARS-CoV-2 Mpro, besides, the previously discussed antiviral activities of the examined marine compounds and as a part of our continuous program to identify potentially active marine natural products^49–54^ with competent antiviral therapeutic activity^55^ and to search for therapeutics combating SARSCoV-2 Mpro,^8–11,56–61^ we decided to examine the anti-SARS-CoV-2 activities of the thirty-four marine compounds (1–34) and propose their mechanism of action as promising SARS-CoV-2 Mpro inhibitors using molecular docking approach, confirm their docking results through applying detailed molecular dynamics calculations, and finally study the structure–activity relationships for the obtained results in order to help scientists in the future discovery, design and synthesis of new effective anti-SARS-CoV-2 therapeutics in the near future.

## 2. Materials and methods

### 2.1. Molecular docking study

Molecular docking studies were performed for the pederins, mycalamides, onnamides and theopederins related compounds (1–34) listed in (Schemes 1–4) against the dimeric form of the Mpro of SARS-CoV-2 using the MOE 2019.012 suite.^62^ Moreover, both the co-crystallized inhibitor (O6K, 35) of the used dimeric protein (6Y2G),^63^ besides the co-crystallized one (N3, 36) extracted from the monomeric protein (6LU7)^64^ of SARS-CoV-2 Mpro were added to the tested database as two reference standards.

#### 2.1.1. Pederins, mycalamides, onnamides and theopederins (1–33) preparation

The ChemDraw professional 17.0 was used to sketch the 2D chemical structures of the selected pederin, mycalamide, onnamide, and theopederin compounds (1–34) which were copied to the MOE window individually. Each transferred compound was converted to its 3D form, energy minimized after the adjustment of its partial charges as well, and saved as (.moe) extension to be ready for the docking step as described earlier.^65–67^ Moreover, the co-crystallized inhibitors of both the used dimeric SARS-CoV-2 Mpro (6Y2G) besides that of the monomeric form (6LU7) (35 and 36, respectively) were extracted and saved separately to be used as reference standards. Finally, all the aforementioned prepared compounds (1–36) were inserted in one database file and saved as (.mdb) extension to be uploaded to the ligand site during the docking process.

#### 2.1.2. The target dimeric Mpro of SARS-CoV-2 preparation

The target dimeric form of Mpro enzyme of SARS-CoV-2 was downloaded from the Protein Data Bank (PDB code: 6Y2G).^63^ Also, it was subjected to correction, 3D protonation, and energy minimization as described before^68–70^ to be ready for the docking step.

#### 2.1.3. Docking of the prepared database (1–36) to the dimeric Mpro of SARS-CoV-2

A general docking process was initiated after uploading the previously mentioned database in place of the ligand and the prepared protein in place of the receptor. The docking site was selected to be the binding site of the co-crystallized α-ketoamide inhibitor (O6K, 35) of the dimeric SARS-CoV-2 Mpro pocket. Also, the general docking specifications were selected to be triangle matcher, London dG, GBVI/WSA dG, and rigid receptor for the placement methodology, first scoring methodology, final scoring methodology, and refinement methodology, respectively.^71–73^ After completion of the docking process, the best pose for each examined compound-according to the score and RMSD values-was selected for further investigations.

**Fig. 1:**
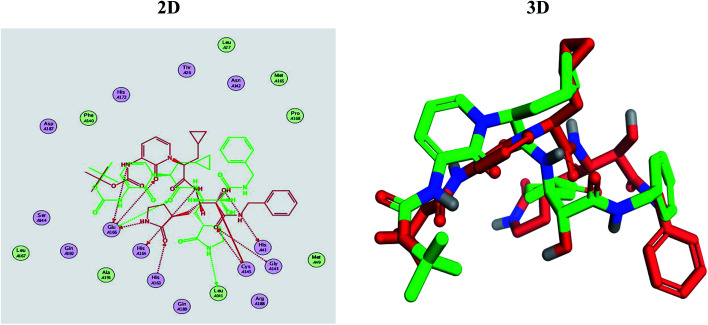
Superimposed poses of the docked O6K inhibitor (represented in green color) over the native co-crystallized one (represented in red color) produced from the redocking process inside the Mpro binding pocket. Left (2D) and right (3D) graphical representations.

It is also worth mentioning that a validation process for the applied MOE program was performed at first through redocking of the co-crystallized α-ketoamide inhibitor (O6K, 35) of the used dimeric Mpro of SARS-CoV-2 at its binding pocket. The valid performance was confirmed by the observed low RMSD values (1.46) describing the root mean squared deviation between the native and redocked poses of the co-crystallized α-ketoamide inhibitor (35) (Fig. 1).^74–76^

### 2.2. Molecular dynamics simulations

Models showing the best docking scores of the most promising leads, as well as reference ligands (O6K and N3) in complex with SARS-CoV-2 Mpro, were chosen as starting coordinates for 200 ns all-atom molecular dynamics simulation using the GROMACS-2019 software package.^77,78^ The symmetry operations (rotations and translations) by the VMD software were applied using the transformation matrices within the PDB crystalline files to obtain the ligand–Mpro dimers. Parameterization of all investigated ligands and generation of their respective topology files were automatically generated using the CHARMM-General Force Field program (Param-Chem project; https://cgenff.umaryland.edu/).^79^ The CHARMM36m force field was preferred for the proteins within all MD simulations.^9,80,81^ Each ligand–Mpro model was solvated within a cubic box of the TIP3P water model under periodic boundary conditions implementation allowing a minimum of 10 Å marginal distance between each 3D box side and the protein.^82^ The residues of the Mpro target protein were assigned at their standard ionization states under physiological conditions (pH = 7.0), while the net charge of the entire systems were neutralized using sufficient numbers of potassium and chloride ions being added *via* the Monte-Carlo ion-placing approach (ESI; Fig. S1†).^83^

The MD simulations were conducted over three conventional stages; one-staged minimization, double-staged equilibration, and production. The minimization step involved initial system geometry optimization through 5 ps (5000 iterations) under the steepest descent algorithm.^8^ The second two-staged equilibration step proceeded for 100 ps (100 000 iterations) per stage. Under a constant number of particles, volume, and temperature (*NVT*) ensemble, the first equilibration was conducted at 303.15 K being regulated by the Berendsen temperature coupling method.^84^ Whereas the second equilibration stage was performed under a constant number of particles, pressure, and temperature (*NPT*) ensemble at 303.15 K and 1 atmospheric pressure regulated by the Parrinello–Rahman barostat method.^85^ A force was constant of 1000 kJ mol^−1^ nm^−2^ was used for preserving original protein folding and restraining all heavy atoms during the minimization and equilibration processes. The production stage involved 200 ns MD simulation runs under constant pressure (NPT ensemble) while using the Particle Mesh Ewald (PME) algorithm for computing the long-range electrostatic interactions.^86^ All covalent bond lengths, including hydrogens, were modeled under the implemented linear constraint LINCS method allowing an integration time step size of 2 fs.^87^ Both Coulomb's and van der Waals non-bonded interactions were truncated at 10 Å using the Verlet cut-off scheme.^88^

Computing comparative analysis tools, including root-mean-square deviation (RMSD) and root-mean-square fluctuation (RMSF), were performed through analyzing the MD trajectories using the GROMACS built-in tools. The difference RMSF (ΔRMSF) was estimated for each ligand-bound protein relative to the SARS-CoV-2 Mpro apo/unliganded state (PDB code: 7C2Q; atomic resolution 1.93 Å), where ΔRMSF = RMSF_apo_ − RMSF_holo_. The same previous preparation, minimization, equilibration, and 200 ns all-atom MD simulation production were applied to the Mpro apo state, except no ligand preparation was performed. The Hydrogen Bond Analysis within Visual Molecular Dynamics ver.1.9.3 software (VMD; University of Illinois, Urbana–Champaign, USA) was utilized to estimate and monitor the number of ligand–Mpro intermolecular hydrogen bonding over the whole simulation periods. The cut-off values for all hydrogen bond (donor–H⋯acceptor) distance and angle were assigned at 3.0 Å and 20°, respectively.^89,90^ Finally, the binding-free energy between the ligand and protein was estimated *via* the Molecular Mechanics/Poisson–Boltzmann Surface Area (MM/PBSA) using the GROMACS “*g_mmpbsa*” module. The MM/PBSA calculations provided more insights regarding the magnitude of ligand–protein affinity, the nature of the interaction, in addition to the residue-wise contributions within the binding-free energy calculations.^91^ Important MM/PBSA parameters for polar/solvation calculations were set at solvent dielectric constant (80 pdie), solute dielectric constant (2 pdie), the radius of solvent probe (1.40 Å), and reference vacuum (1 vdie). Concerning SASA apolar solvation; the radius of SASA solvent probe, offset constant, and solvent surface tension were set at 1.40 Å, 3.8493 kJ mol^−1^, and 0.0227 kJ mol^−1^ Å^−2^, respectively. Finally, parameters for the continuum-integral-based model were set as solvent probe radius 1.25 Å, bulk solvent density (0.0334 Å^−3^), and 200 for numbers of quadrature points per Å^2^. The MM/PBSA calculations of all simulated systems were applied on representative frames for the whole MD simulation runs (200 ns). Using the GROMACS command-lines “*gmx trjcat*”, four hundred representative snapshots/frames were spared out of the whole trajectory file at specified time intervals (*i.e.* one frame/snapshot every 500 ps). For representing the ligand–protein conformational analysis across specific timeframes, the Schrödinger™ Pymol™ graphical software ver. 2.0.6 was used.^92^

## 3. Results and discussion

### 3.1. Docking studies

Analyzing the binding modes of the co-crystallized inhibitors (35 and 36) of the dimeric and monomeric forms of SARS-CoV-2 Mpro showed an asymmetric binding in each case. Furthermore, molecular docking of the pederins, mycalamides, onnamides and theopederins related compounds (1–34) against the dimeric form of SARS-CoV-2 Mpro achieved very promising results to be discussed in detail. Generally, the binding score order of the tested compounds was found to be in the following order: onnamides > theopederins > pederins > mycalamides. Many compounds were found to be superior to the co-crystallized inhibitor of the dimeric form (35) especially those of the onnamides family (9–19, 21). It was also noted that the co-crystallized inhibitor of the monomeric form (36) got a binding score of −10.24 kcal mol^−1^ which was better than that of the dimeric form (35) which recorded a binding score of −8.77 kcal mol^−1^ (Table 1).

**Table 1:** The binding scores of the tested pederins, mycalamides, onnamides and theopederins related compounds (1–34) besides the docked co-crystallized inhibitors (35 and 36) against the dimeric form of SARS-CoV-2 Mpro pocket

No.	Compound	Score^a^
**Pederins family**		
1	Pederin	−7.95
2	Pesudopederin	−7.16
3	Pederone	−7.86

**Mycalamides family**		
4	Mycalamide A	−7.89
5	Mycalamide B	−7.51
6	Mycalamide C	−7.09
7	Mycalamide D	−7.38
8	Mycalamide E	−7.57

**Onnamides family**		
9	Onnamide A	−9.50
10	13-Des-*O*-methyl onnamide A	−9.20
11	Dihydro-onnamide A	−10.19
12	Onnamide B	−9.28
13	17-Oxo-onnamide B	−9.21
14	Onnamide C	−9.60
15	Onnamide D	−9.40
16	Onnamide E	−9.54
17	Pseudo-onnamide A	−9.81
18	Dihydro-oxo-onnamide A	−9.41
19	Oxo-onnamide A	−9.47
20	Onnamide F	−8.61
21	Z-onnamide A	−9.06

**Theopederins family**		
22	Theopederin A, (α-OH)	−8.24
23	Theopederin A, (β-OH)	−8.18
24	Theopederin B	−7.89
25	Theopederin C	−7.93
26	Theopederin D	−8.32
27	Theopederin E	−7.12
28	Theopederin F	−7.99
29	Theopederin G	−8.45
30	Theopederin H	−8.11
31	Theopederin I	−8.43
32	Theopederin J	−8.13
33	Theopederin K	−7.92
34	Theopederin L	−8.06

**Co-crystallized inhibitor**		
35	6Y2G (O6K)	−8.77
36	6LU7 (N3)	−10.24

a Score unit is (kcal mol^−1^).

Regarding the docking results depicted in Table 1, we decided to further study pederin (1) as the most promising member of pederins, dihydro-onnamide A (11), onnamide C (14), and pseudo-onnamide A (17) as the most promising members of onnamides, and theopederin G (29) as the most promising member of theopederins as well, besides the two docked co-crystallized inhibitors (35 and 36) as represented in Tables 1 and 2. Also, the 2D binding interactions of the aforementioned compounds were depicted in (ESI; Fig. S2†).

**Table 2:** 3D binding and positioning of the further five examined marine products (1, 11, 14, 17, and 29) besides the docked co-crystallized inhibitors (35 and 36) towards the binding pocket of SARS-CoV-2 Mpro^a^

Comp.	3D pocket binding	3D positioning
Pederin (1)	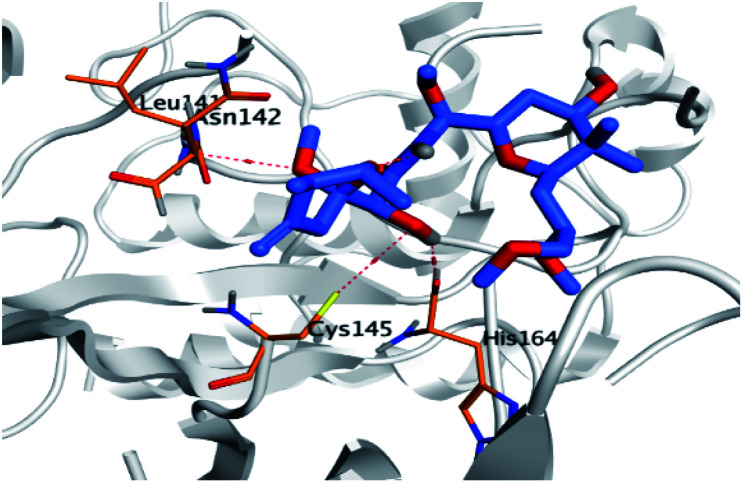	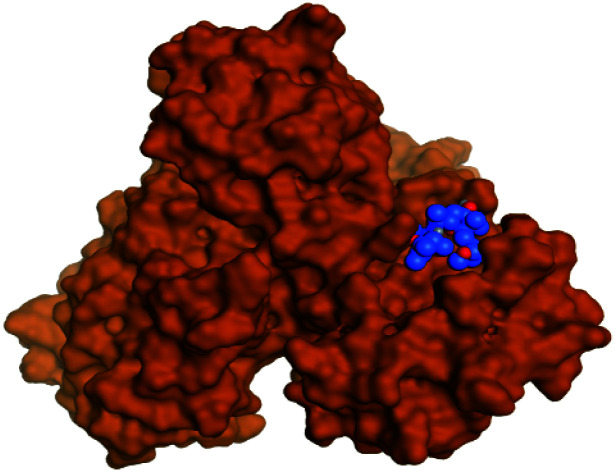
Dihydro-onnamide A (11)	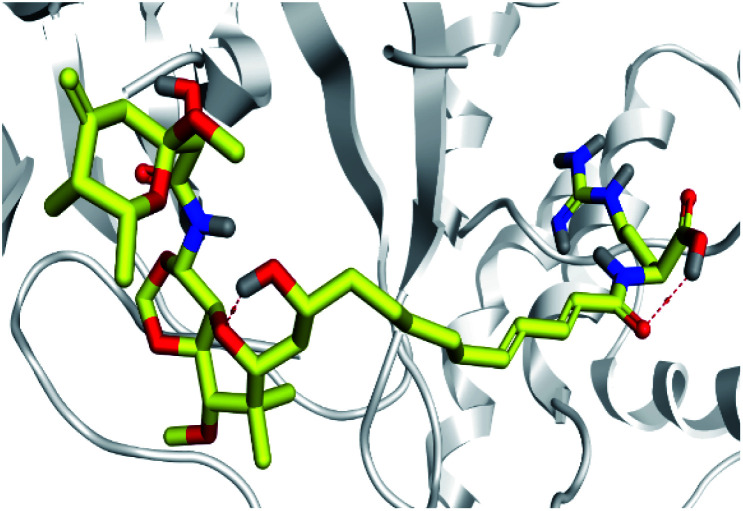	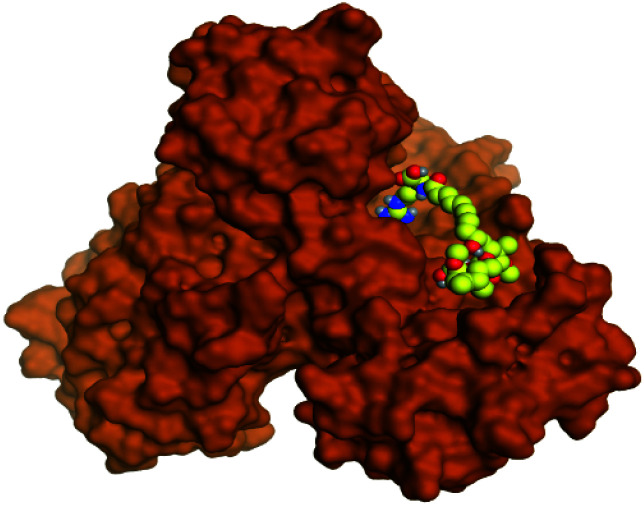
Onnamide C (14)	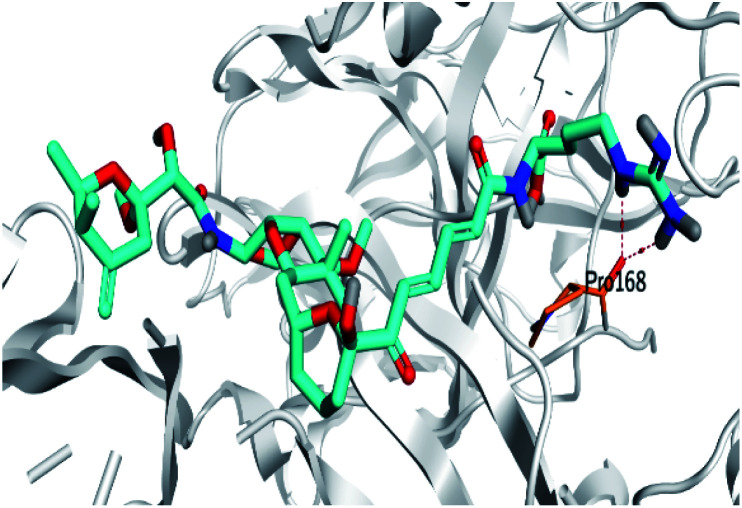	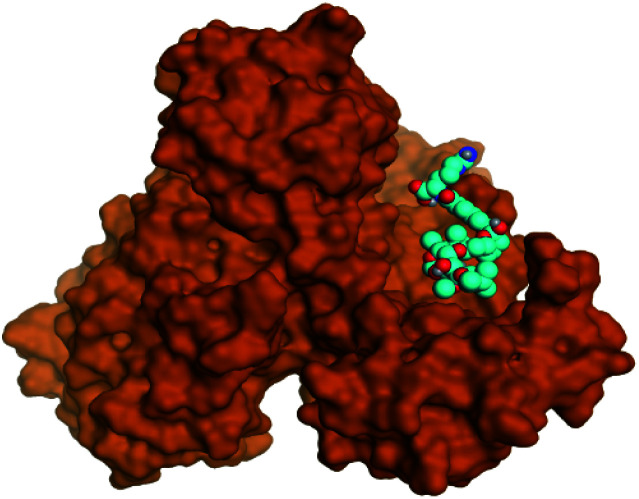
Pseudo-onnamide A (17)	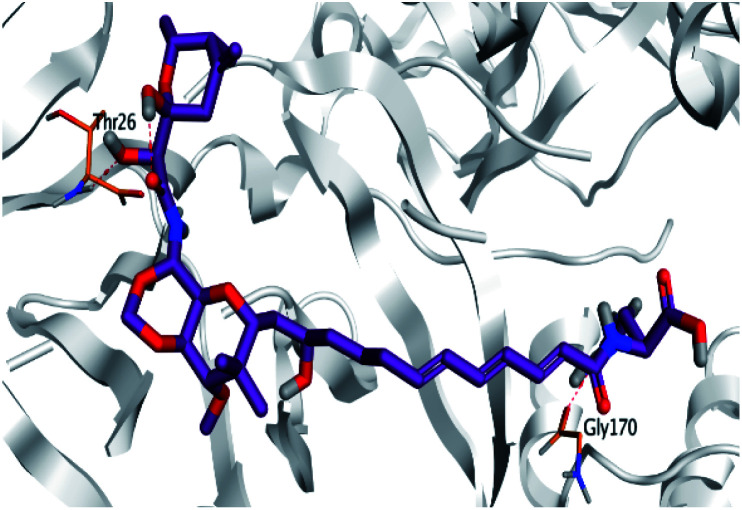	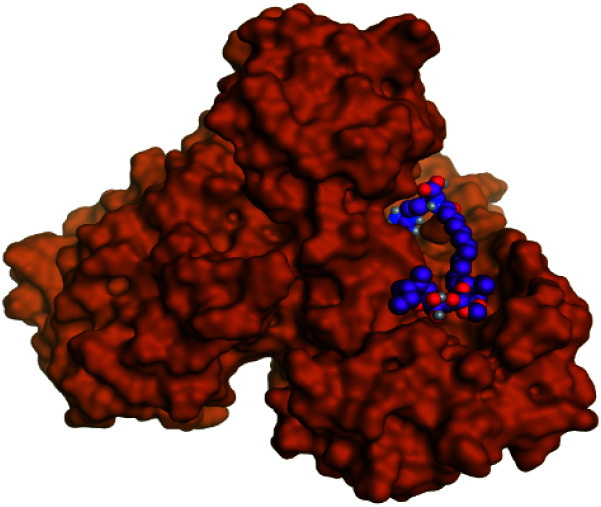
Theopederin G (29)	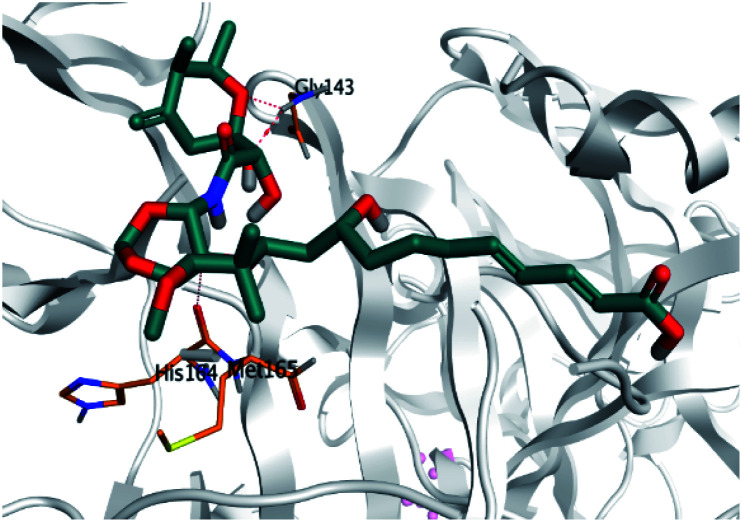	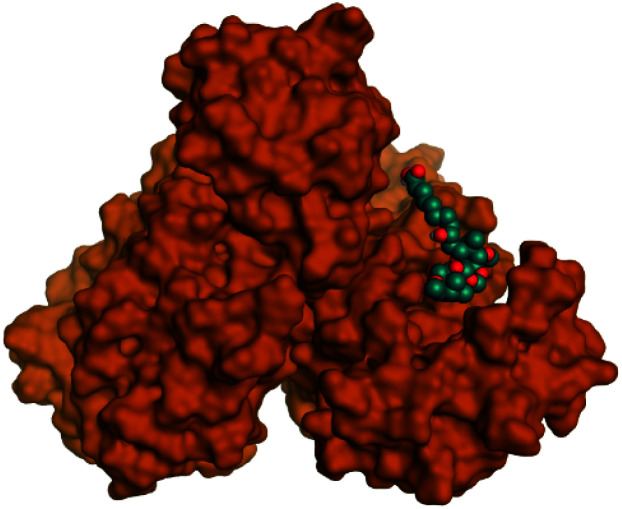
6Y2G co-crystallized inhibitor (O6K, 35)	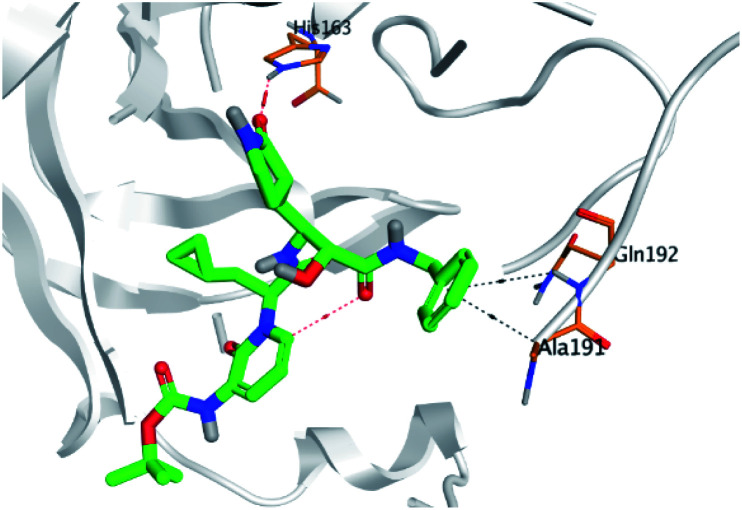	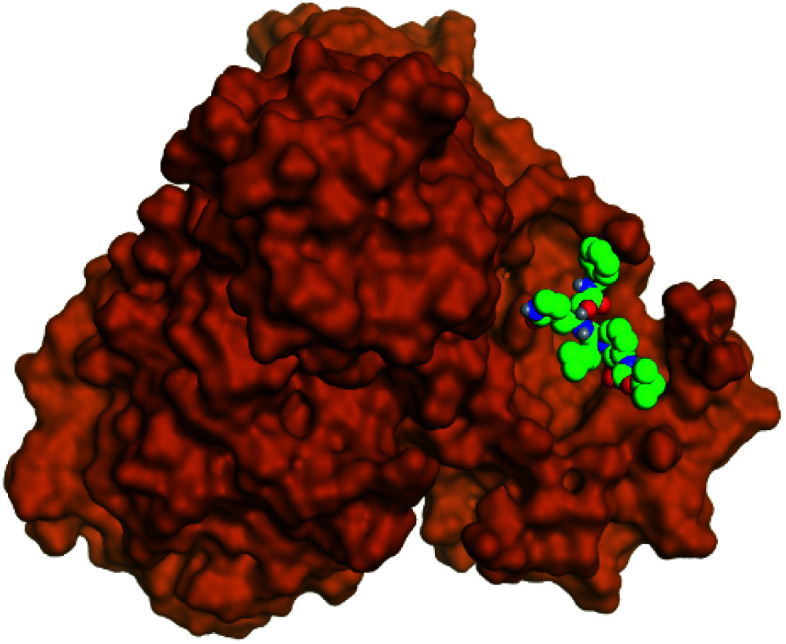
6LU7 co-crystallized inhibitor (N3, 36)	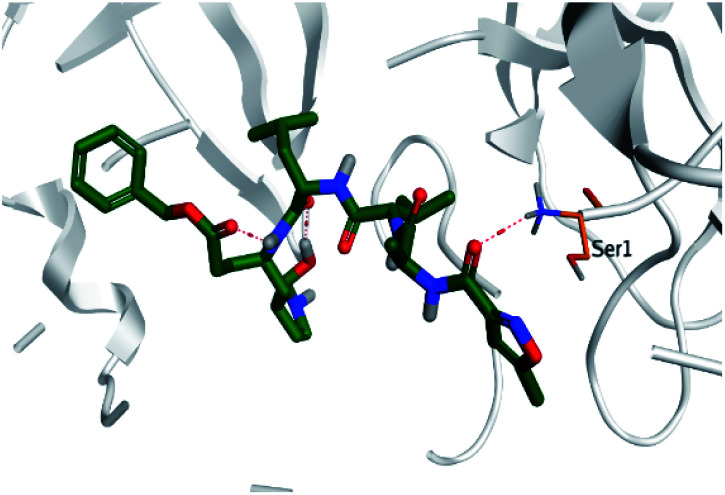	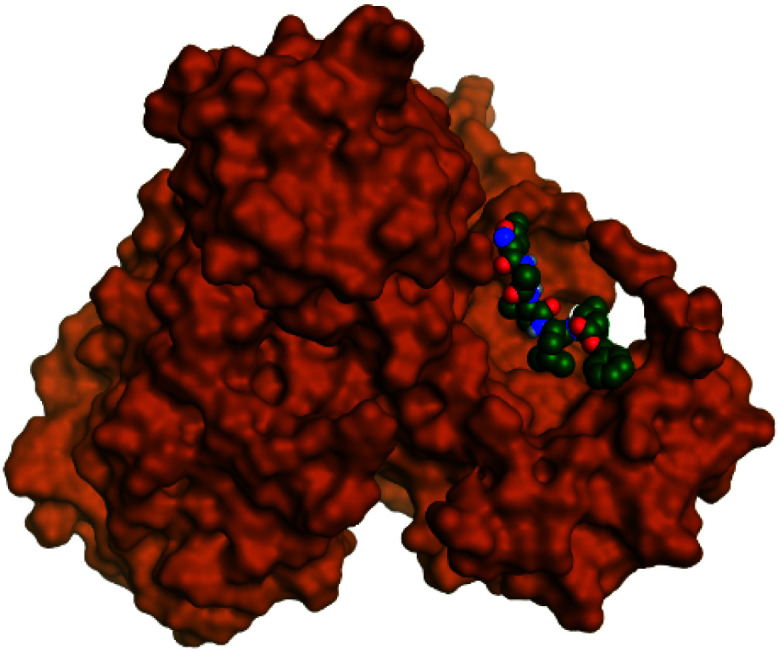

a The red dash represents H-bonds and the black dash represents H-pi interactions.

Regarding both Tables 1 and 2; the co-crystallized inhibitor of the dimeric SARS-CoV-2 Mpro (O6K, 35) was found to form one H-bond with His163 at 2.98 Å and two pi–H bonds with Ala191 and Gln192 at 3.64 and 4.58 Å, respectively. On the other hand, the co-crystallized inhibitor of the monomeric SARS-CoV-2 Mpro (N3, 36) got stabilized inside the binding pocket of the dimeric SARS-CoV-2 Mpro through the formation of only one H-bond with Ser1 at 3.29 Å.

Pederin (1) as the most promising member of pederins showed a binding score of −7.95 kcal mol^−1^ and formed three H-bonds with His164, Asn142, and Cys145 at 2.94, 3.17, and 3.50 Å, respectively. Moreover, the binding modes of dihydro-onnamide A (11), onnamide C (14), and pseudo-onnamide A (17) as the most promising members of onnamides were studied in detail. Dihydro-onnamide A (11) achieved a binding score of −10.19 kcal mol^−1^ which was found to be the best one among all the tested marine compounds. Also, surprisingly it achieved this superior binding strength without forming any bonds with the pocket amino acids which indicated its great stability inside the binding pocket of SARS-CoV-2 Mpro. However, onnamide C (14) binding score was found to be −9.60 kcal mol^−1^ with the formation of two H-bonds with Pro168 at 3.06 and 3.07 Å through its guanido group. On the other hand, pseudo-onnamide A (17) got stabilized inside the pocket through the formation of two H-bonds with Gly170 and Thr26 at 3.04 and 3.05 Å, respectively, with a binding score of −9.81 kcal mol^−1^. Theopederin G (29) as the most promising member of theopederins gave a binding score of −8.45 kcal mol^−1^. Also, it formed three H-bonds, two with Gly143 and one with His164 amino acids, at 2.93, 3.04, and 3.44 Å, respectively.

Collectively, the aforementioned results referred to very promising binding scores and interactions which indicate the expected promising intrinsic activities of the tested marine compounds at the same time.

### 3.2. Molecular dynamics simulations

Being an effective tool for investigating the relative stability of ligand–target complex as well as their respective dynamic behavior, MD simulation studies were performed. The latter computational tool is considered particularly beneficial for exploring the conformation space of ligand–target complex being more efficiently than other *in silico* tools including molecular docking and mechanics energy minimization approaches for just static image analysis.^93^ Showing relevant ligand–Mpro docking interactions, the top docked poses of the investigated compounds related to different families of the studied polyketides (pederin (1, D1), dihydro-onnamide A (11, D2), onnamide C (14, D3), pseudo-onnamide A (17, D4), and theopederin G (29, D5)), besides the two reference ligands (N3 and O6K) within the SARS-CoV-2 Mpro canonical binding site were subjected to 200 ns all-atom MD simulation.

#### 3.2.1. Stability analysis of ligand–protein complexes

Throughout the 200 ns all-atom MD runs, several examined agents illustrated significant global stability within the target's canonical binding site as being confirmed through the monitored RMSD trajectories. Generally, RMSD estimates the molecular deviation of a particular ligand relative to a designated original/reference structure. Such an analytical tool would provide a good indication for the ligand–target stability and the adopted MD simulation protocol was valid. Target's instability and significant conformational alterations are associated with high RMSD trajectories.^94^ On the other hand, high complex RMSD would correlate to limited ligand–target affinity where the ligand is unable to be confined within the target's canonical binding site along the simulation periods.^95^

**Fig. 2:**
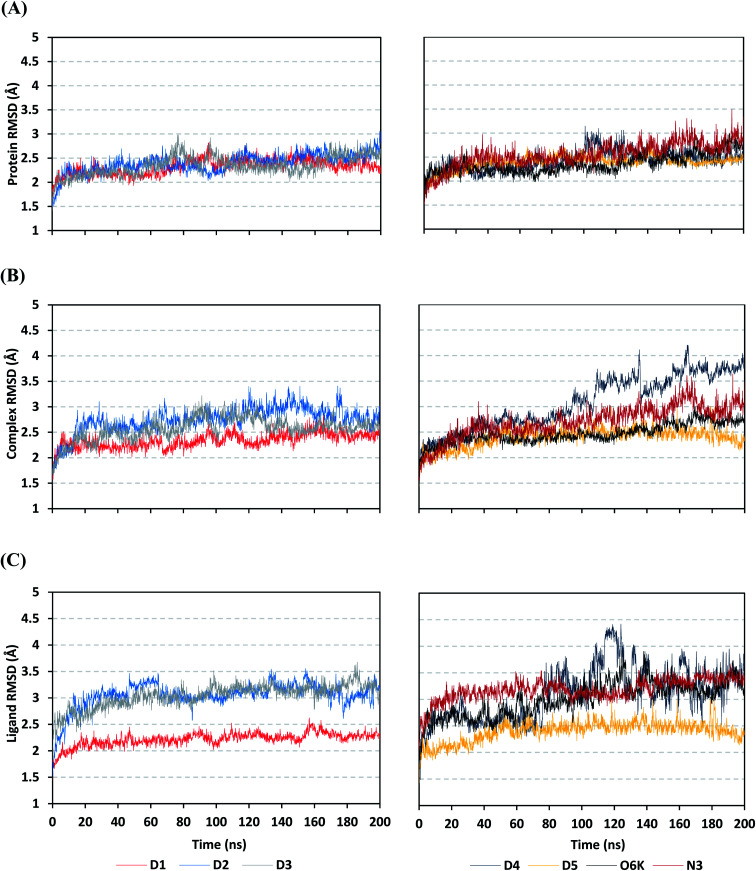
Stability analysis of generated RMSD trajectories for investigated compounds and reference ligands in complex with SARS-CoV-2 Mpro protein along 200 ns all-atom MD simulation. (A) Protein C-α RMSD; (B) complex backbone RMSD; (C) sole ligand backbone RMSD trajectories (Å), across MD simulation time (ns).

The estimated RMSD deviations for Mpro proteins, in reference to their respective alpha-carbon atoms (C-α RMSD), depicted an overall typical behavior for MD simulations (Fig. 2A). Over the initial frames, the protein's C-α RMSD tones increases as a result of constraining release at the beginning of MD simulation runs. Following the first 20 ns of the MD runs, steady protein's C-α RMSD trajectories were obtained for more than half of the simulation run time (>150 ns), except for minimal fluctuation for D4 and N3-bound protein around 100–200 ns and near the end of MD simulation runs, respectively. Notably, almost all investigated ligands leveled off at comparable RMSD trajectories across the trajectory plateau and till the end of MD simulation courses (D1 2.32 ± 0.16 Å, D2 2.39 ± 0.22 Å, D3 2.35 ± 0.20 Å, D5 2.39 ± 0.20 Å, and O6K 2.36 ± 0.20 Å). Slightly higher values assigned for D4 and N3 (2.43 ± 0.22 Å and 2.55 ± 0.26 Å, respectively) were correlated to the depicted fluctuations across the MD simulation timeframes. On the other hand, the D1-bound Mpro systems managed to exhibit the steadiest C-α RMSD tones, exhibiting the lowest standard deviation value after the equilibration was attained. The described dynamic behavior of the investigated Mpro proteins indicates the successful convergence of the target proteins. Moreover, the above-depicted protein's C-α RMSD tones also infer that successful system minimization, relaxation, and thermal equilibration stages have been adopted before the MD production step and thus, no further extension of the MD simulation beyond the 200 ns period was needed.

For gaining more insights concerning the ligand's confinement within the Mpro canonical binding site across the MD run, the RMSD fluctuations were monitored for the combined ligand–protein complex in reference to the protein backbone initial frame (Fig. 2B). Despite limited fluctuations, the binary complexes of Mpro with almost all examined compounds and reference ligands managed to reach their respective dynamic equilibrium illustrating backbone RMSD plateau, beyond the 30 ns, indicating sufficient complex stability. Despite the differential backbone RMSD tones at the initial MD simulation frames, the investigated complexes managed to converge along the last 200 ns reaching a final RMSD around 2.54 ± 0.17 Å. Nevertheless, this dynamic behavior was not the same for the D4–Mpro complex since beyond 90 ns high fluctuations were depicted (∼3.51 ± 0.27 Å) till the end of the MD simulation run indicating significant ligand orientation shift. Interestingly, compounds D1, D5, and crystalline reference O6K achieved early equilibration with the steadiest complex RMSD trajectories and low comparative average values (2.33 ± 0.15 Å, 2.39 ± 0.19 Å, and 2.43 ± 0.18 Å, respectively) as compared to other ligands (∼2.67 ± 0.37 Å). The latter observation highlights the better ligand's retainment for D1 and D5 within the protein pocket as compared to the rest of examined compounds as well as the reference N3 potent inhibitor.

Being considered as an additional descriptor for ligand-pocket confinement and convergence of the simulated proteins, the sole ligand RMSDs relative to the reference protein backbone frame were monitored along the MD simulation runs (Fig. 2C). Lower average RMSD trajectories were assigned to the above suggested stable ligands, D1 (2.22 ± 0.13 Å), D5 (2.39 ± 0.18 Å), and O6K (2.46 ± 0.20 Å), as compared to D2 (3.05 ± 0.25 Å), D3 (3.04 ± 0.22 Å), and N3 (3.18 ± 0.20 Å). In concordance with the above D4-Mpro complex RMSD trajectories, higher fluctuations and average ligand RMSD trajectories were assigned for D4 (average 4.09 ± 0.81 Å) ensuring its significant orientation shift following the 90 ns of MD run. It is worth mentioning that protein RMSD trajectories were within 1.5-fold those of their respective ligands, with higher values for D4, the thing that further confirms the successful convergence of the ligand–protein complexes inferring the suitability of 200 ns simulation timeframe needing no further MD extension.

The MD simulation convergence was further validated and monitored *via* Principal Component Analysis (PCA). The latter approach examines the protein's collective dynamic motion and behavior out of the MD trajectories through constructing and diagonalizing covariance matrix from protein's C-α atomic coordinates.^96^ The average covariance matrix allows capturing strenuous atom motions throughout both the eigenvectors and eigenvalues. Typically, the covariance matrix eigenvectors elucidate the atom's overall motion direction, while the eigenvalues represent the atom-wise contribution values within such motion. In these regards, both the covariance matrix eigenvectors and eigenvalues furnish the modes of collective motion and their respective amplitudes. The GROMACS “*gmx_covar*” command script was used for constructing and diagonalizing the covariance matrices, whereas, “*gmx_anaeig*” was used for presenting the most dominant modes (eigenvectors-1 and -2) in addition to estimating the overlap between principal components and trajectory coordinates. Since the corresponding eigenvalues indicate the dynamic behavior and degree of fluctuations, covariance matrix with lower trace values would correlate with the minimal escalation of collective protein motion which further denote the MD simulation convergence.^97–99^ Applying the PCA technique on the last 50 ns MD trajectories while comparing it with that for the rest of MD simulation frames would be fundamental for monitoring and validating the MD simulation convergence.

**Fig. 3:**
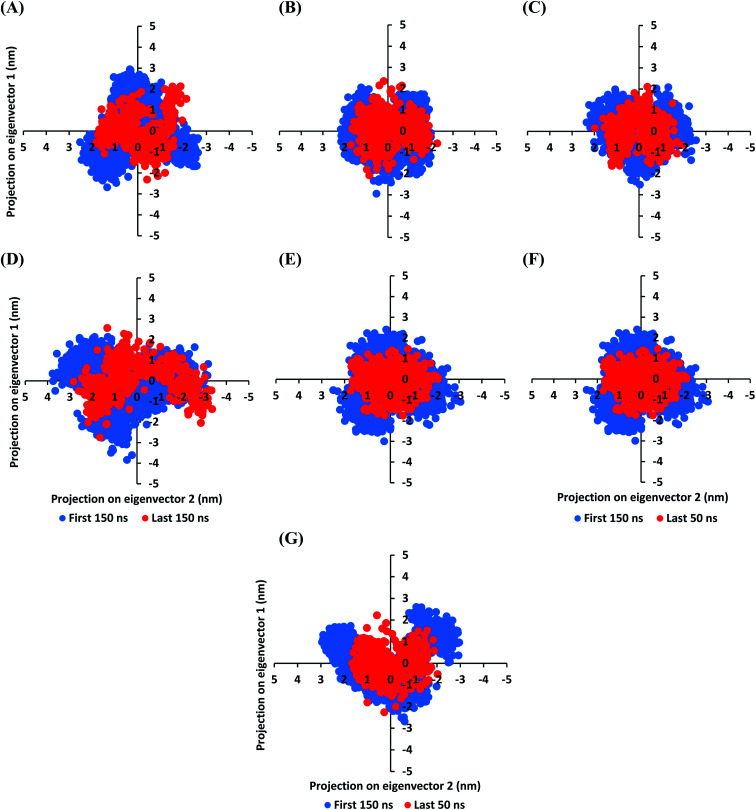
Projections of Mpro atoms in phase space along the first two dominant eigenvectors (eigenvector-1 and -2). (A) D1; (B) D2; (C) D3; (D) D4; (E) D5; (F) O6K; (G) N3-bound Mpro proteins. The PCA calculations were conducted cross initial 150 ns and last 50 ns MD simulation trajectories exhibiting differential expected structural stability and convergence.

Interestingly, the average trace value of the covariance matrix at the last 50 ns was of lower magnitudes as compared to that along the first 150 ns MD simulation trajectories (Fig. 3). The average trace value of the covariance matrix for almost all investigated models showed a nearly 30% decrease for the MD trajectories at the last 50 ns (Table 3). The latter ensures higher stability of the protein atoms at the last 50 ns which in turn conferring a validated convergence of the adopted MD simulation. On the other hand, only the D4 model exhibited ∼15% reduction which came in good agreement with the depicted fluctuations observed at its previously described C-α RMSD trajectories. Concerning comparative PCA analysis, it was noticed that comparable MD convergence patterns were assigned for the Mpro proteins in complex with D1, D2, D5, and O6K (average 7.419155 ± 0.24 and 5.45171 ± 0.18 nm^2^ for initial 150 ns and last 50 ns frames, respectively). The lowest magnitude was obtained for the D1 system at the last 50 ns correlating with the observed steady C-α RMSD following equilibration being superior over other investigated systems (Fig. 2A). Both D3 and N3 showed higher covariance matrix traces at both investigated MD trajectory frames as compared to those for the above stably converged models. Nevertheless, both systems showed the same magnitude of reduction for their respective average trace value of the covariance matrix being around 30%. Based on the above findings, validated MD simulation convergence was obtained for the above-investigated models ensuring the adequacy of the 200 ns MD simulation timeframe for exploring the ligand–Mpro thermodynamic behaviors.

**Table 3:** The PCA approach for monitoring and validating the MD simulation convergence for the investigated ligand-bound Mpro proteins

Ligand-bound Mpro protein	Trace of the covariance matrix at selected MD trajectories (nm^2^)	
	First 150 ns	Last 150 ns
D1	7.41563	5.21810
D2	7.67162	5.40688
D3	8.17157	6.67493
D4	9.54521	8.11823
D5	7.08523	5.54116
O6K	7.50414	5.64070
N3	8.25667	6.90142

Since both the RMSD analysis and PCA techniques highlight the significant ligand–target stability for several examined ligands, it was beneficial to further investigate the local protein flexibility and how this could be contributed to the ligand binding. The fluctuation of the target's residues was monitored by estimating the RMSF stability validation parameter which was able to highlight the residue-wise contribution within the target protein stability. Typically, RMSF provides a valuable evaluation of the target's residues dynamic behavior represented as both fluctuation and flexibility, through estimating the average deviation of each protein's amino acid concerning its respective reference position across time.^100^ Within the presented manuscript, the difference root-mean-square fluctuation (ΔRMSF) was a better estimation of the protein local flexibility being the RMSF difference for each ligand-bound protein relative to the SARS-CoV-2 Mpro apo state (ΔRMSF = apo RMSF − holo RMSF). A ΔRMSF cut-off value of 0.30 Å was relevant for estimating the significant alterations within the protein's structural movements meaning that amino acids with ΔRMSF above 0.30 were considered of limited mobility. This adopted cut-off was able to identify the immobile residues, whereas excluding those exhibiting inherited flexibility including those at flexible protein secondary structure (loops) and terminal segments.^89,101^ Investigating the RMSF trajectories essentially execute for a trajectory region considered stable. Based on the above protein's C-α RMSD analysis (Fig. 2A), the Mpro proteins target were of significant conformational stability along the 200 ns MD simulations for all systems (<3.5 Å), despite the limited fluctuations for D4 and N3 systems. Therefore, the C-α RMSF calculations were reasoned for estimation across the whole MD simulation trajectories.

**Fig. 4:**
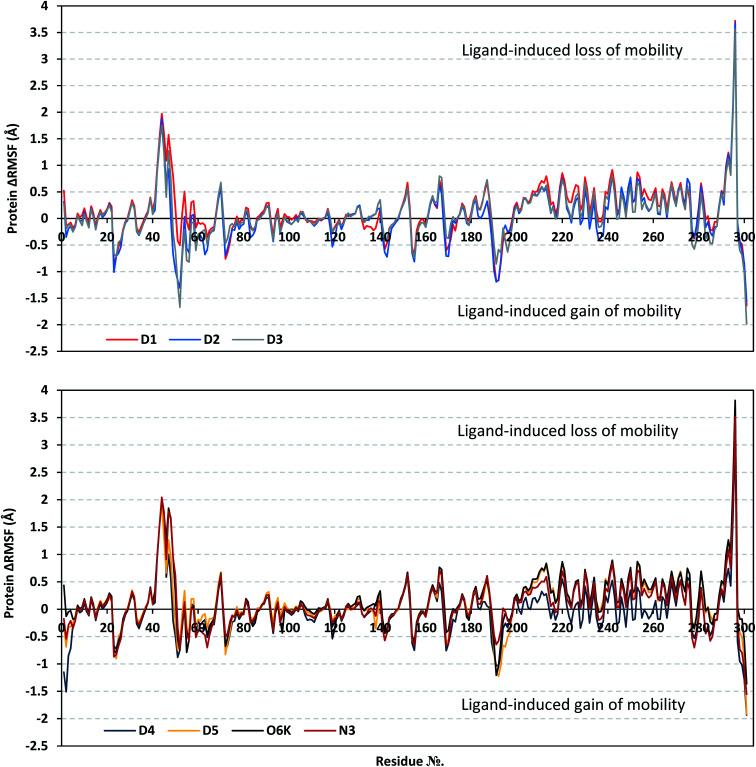
Analysis of ΔRMSF trajectories *versus* residue number for Mpro protein, in complex with isolated compounds and reference ligands, throughout the whole MD simulation window. The ΔRMSF values, in reference to protein C-α atoms, were estimated considering independent MD simulation of Mpro apo/unliganded state (PDB code: 7C2Q; atomic resolution 1.93 Å) against the holo ones being complexed with the investigated isolated ligands or references, O6K and N3. The ΔRMSF trajectories are represented as a function of residue number (residues 1-to-303).

Throughout the ΔRMSF analysis, the free terminals residues showed a typical fluctuation pattern with the highest negative ΔRMSF values in comparison to the core residues (Fig. 4). This behavior is highly reasoned since these terminal residues are most likely to fluctuate at the highest deviations in comparison to core residues the thing that is typical for a well-behaved MD simulation. Interestingly, higher fluctuation patterns were depicted for the residues of each ligand–protein complex at the Mpro C-terminus as compared to those located near the NH_2_ end (average −1.32 ± 0.46 *versus* −0.26 ± 0.58 Å). The terminal flexible residues are at regions located >15 Å from the protein's canonical binding site. The latter infers to the ability of the active site to accommodate bulkier ligands. Several distinct residue ranges including; 41–48, 162–167, 185–188, and 203–296, illustrated significant immobility possessing an average ΔRMSF above the 0.30 Å threshold. Notably, the residue range 289–296 being vicinal to the protein's C-terminal showed one of the highest immobility profiles (ΔRMSF up to 3.81 ± 0.13 Å). Such dynamic behavior confers significant influence of ligand's binding upon the stability of these C-terminal vicinal residues which came in great agreement with reported studies.^102^

Concerning comparative protein's local stability, lower ΔRMSF trends were assigned for D4-bound Mpro residues relative to those of the other isolated and reference ligands. This was recognized across several ranges of protein residues, most notably for the 203–270 residue range. On the other hand, the high stability of D1, D5, and O6K-bound proteins confirmed with the presented ΔRMSF findings which further confirms the superior stability of these three latter systems as being previously discussed *via* RMSD and PCA data.

#### 3.2.2. Local protein flexibility and fluctuation of target's residues

For gaining more insights regarding the ligand interactions with Mpro pocket, a comparative analysis of the furnished ΔRMSF trajectories has proceeded regarding the specific flexibility of Mpro key lining pocket residues. Interestingly, several canonical pocket residues depicted significant immobility with ΔRMSF above the cut-off mobility threshold 0.30 Å (Table 4). Residues lining the S1′ subsite showed significant mobility with the lowest of all ΔRMSF values (lots of negative values) the thing that infers the great mobility indices of such residues. However, only the catalytic His-41 showed limited flexibility only for D1 and O6k reference ligand (ΔRMSF = 0.34 Å and 0.33 Å, respectively), while being at the marginal cut-off for D3 (0.29 Å) and D5 (0.29 Å). The other catalytic residue, Cys145, exhibited significant mobility with all ΔRMSF being of negative values. It is worth mentioning that residues being vicinal to His41 (Pro39 and Val42-to-Asp48) exhibited significantly high RMSF trajectories (0.37 Å and up to 2.04 Å) for the investigated complexes. Notably, the D1, O6K, and N3 systems illustrated the highest immobility profiles for the above His41-vicinal residues (average ΔRMSF = 1.28 ± 0.49 Å, 1.43 ± 0.53, and 1.38 ± 0.57 Å, respectively). Thus, it was suggested that the ligand-His41 hydrogen bond pair has a much more pronounced impact on ligand–protein stability over that of the other catalytic residue, Cys145. This was in great concordance with our previous study investigating promising natural scalaranes sesterterpenes isolated from the Red Sea marine sponge *Hyrtios erectus* as promising inhibitors of SARS-CoV-2 Mpro target.^77^

**Table 4:** Estimated ΔRMSF^a^ values for investigated ligand–Mpro proteins along the whole MD simulation

Binding site subsite	Comprising residue	D1	D2	D3	D4	D5	O6K	N3
S1′	**His41**	***0.34***	0.15	0.29	0.12	0.28	***0.33***	0.21
	Gly143	−0.47	−0.72	−0.41	−0.38	−0.41	−0.37	−0.44
	Ser144	−0.18	−0.42	−0.21	−0.19	−0.15	−0.15	−0.16
	Cys145	−0.12	−0.19	−0.12	−0.14	−0.11	−0.09	−0.11
S1	**Phe140**	0.17	0.19	***0.35***	0.27	0.28	***0.33***	0.15
	Leu141	−0.28	−0.34	−0.13	−0.19	−0.15	−0.17	−0.33
	Asn142	−0.58	−0.64	−0.43	−0.41	−0.43	−0.44	−0.57
	**His163**	***0.40***	***0.39***	***0.43***	***0.38***	***0.39***	***0.44***	***0.40***
	**Glu166**	***0.57***	***0.47***	***0.77***	***0.32***	***0.63***	***0.72***	***0.62***
S2	**Met49**	***0.79***	−0.68	−0.28	−0.22	0.27	***0.94***	***0.94***
	**Tyr54**	***0.51***	0.04	−0.21	0.26	***0.34***	0.07	0.13
	**His164**	0.26	0.28	***0.30***	0.27	0.27	***0.34***	0.27
	**Asp187**	***0.69***	***0.33***	***0.73***	0.05	***0.53***	***0.52***	***0.61***
	**Arg188**	**0.35**	0.04	***0.39***	0.03	0.24	0.29	***0.32***
S3	**Met165**	0.19	0.22	***0.32***	0.14	0.21	0.31	***0.30***
	**Leu167**	***0.57***	***0.47***	***0.77***	***0.32***	***0.63***	***0.72***	***0.62***
	Gln189	−0.38	−0.37	−0.02	−0.62	−0.22	−0.17	−0.06
	Thr190	−0.84	−0.99	−0.51	−0.82	−0.80	−0.63	−0.47
	Gln192	−1.17	−1.16	−0.58	−1.03	−1.22	−1.04	−0.58

a Relative difference root-mean-square fluctuation (ΔRMSF) ± standard deviation was estimated for each ligand-associated Mpro protein relative to the SARS-CoV-2 Mpro apo/unliganded state (PDB code: 7C2Q; atomic resolution 1.93 Å). Residues showing significant immobility (ΔRMSF > 0.30 Å) are written in bold and values are in bold italic.

Moving towards Mpro S1 subsite, significant-high ΔRMSF values were depicted across the investigated ligand-bound proteins regarding a couple of pocket lining residues (Phe140, His163, and Glu166). Significant immobility for the Glu166 (from 0.48 and up to 0.80 Å) ensures the reported data within the current literature suggesting the crucial role of S1 subsite Glu166 residues for stabilizing several drug-like and peptidomimetic ligands at the Mpro active site.^8,9,63,77,103–106^ It is worth mentioning that the stability profiles of Phe140 were only associated with D3 (0.35 Å) and O6K (0.33 Å) highlighting the significant role of hydrophobic interactions in stabilizing both ligands at the Mpro binding site. Finally, almost all residues lining the S2 sub-pocket and a couple of residues comprising the S3 one (Met165 and Leu167) showed high trends of significant immobility and limited fluctuations ranging from 0.32 Å and up to 0.94 Å. Interestingly, this residue-wise immobility trade was of the highest positive numbers for the S2 Met49 residue, particularly at the D1, O6K, and N3-bound proteins. Several vicinal residues for the S2 subsite depicted significant rigidity. These immobile residues include Phe185 and Val-186 inferring the stability of ligands within these two respective protein subsites. In brief, the provided ΔRMSF findings highlighted the key role of several S2 amino acids in addition to S3 Met165/Leu167, S1 His163/Glu166, S1′ catalytic His41, as well as vicinal residues for stabilizing the investigated compounds and both reference ligands within the Mpro canonical pocket. Additionally, the ΔRMSF trajectories positively add to suggested sustained stability and compactness of the ligand–Mpro investigated complexes, particularly for D1, across the all-atoms MD simulations. All these came in high concordance with the above presented dynamic behaviors presented by the RMSD and PCA findings.

#### 3.2.3. Conformational analysis and intermolecular hydrogen bonding of ligand–Mpro complexes

**Fig. 5:**
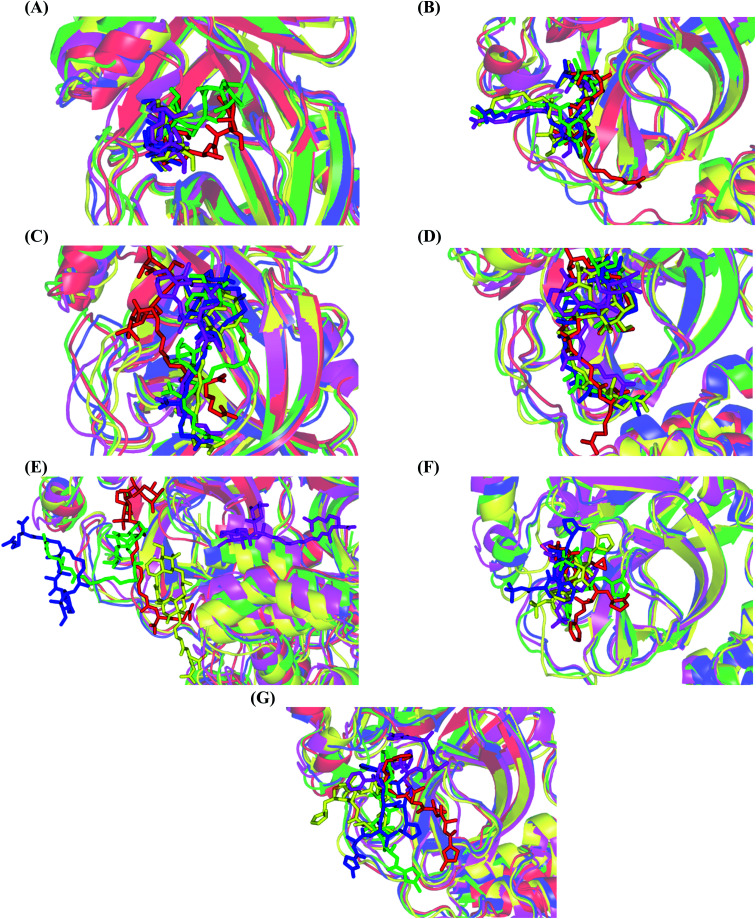
Conformational analysis of ligand–Mpro complex across selected trajectories. (A) D1; (B) D5; (C) D2; (D) D3; (E) D4; (F) O6K; (G) N3. Protein is represented in red, green, blue, yellow, and magenta cartoon 3D-representation corresponding to 0 ns, 50 ns, 100 ns, 150 ns, and 200 ns extracted frames, respectively. The ligands (sticks) are all presented in colors corresponding to their respective extracted frame.

Analysis of key conformational alterations across the MD simulation timeframe was performed through examining the ligand–Mpro models at trajectories of regular intervals. Selected frames at 0, 50, 100, 150, and 200 ns for each ligand–protein model were extracted and minimized to a 0.001 kcal mol^−1^ A^−2^ gradient using the MOE system preparation package. A stable binding profile was assigned for almost all isolated compounds as well as reference ligands. Notably, D1 showed the most limited conformational changes across the selected trajectories, particularly following the 100 ns MD simulation time frame where the ligand exhibited comparable spatial orientation and pocket confinement (Fig. 5A). This could be reasoned as D1 exhibited a lower number of rotatable bonds, particularly since it lacks the long aliphatic tail, the thing that could significantly impact its special stability within the Mpro pocket. Similar behavior was depicted for D5 where comparable spatial orientations were depicted following the 50 ns simulation time with the ligand's tail substitution being directed towards the pocket's solvent-exposed side (Fig. 5B). Despite having a tail substitution, this theopederin family member (D5) is with reasonable rigidity since the tail is being unsaturated with two double bonds. Both depicted D1 and D5 preferential stability came in great agreement with the previously described RMSD findings.

Concerning the onnamides family members, both D2 and D3 exhibited higher aliphatic extensions with reasonable conformational changes being limited to their respective aliphatic tails rather than their tetrahydropyran rings (Fig. 5C and D). However, both ligands showed reasonable confinement within the Mpro pocket site despite their inherited conformational flexibility. Higher tail-related conformational alterations were assigned for D2 rather than D3 since the latter exhibited tail rigidification owing to possessing an additional tetrahydropyran ring due to hydroxyl group-mediated tail cyclization. Ligand confinement within Mpro pocket was not depicted for the other close related onnamide member where D4 abandoned the Mpro canonical binding site at the middle and end of the MD simulation (Fig. 5E). The latter dynamic behavior explains the exhibited higher fluctuations within the complex and sole ligand RMSDs beyond the 90 ns MD simulation time frame. It was suggested that the presence of higher unsaturation (three double bonds conjugation) within the D4 tail could limit its conformational flexibility while limiting its favored maneuvers towards the significant pocket residues. Exhibiting unfavoured tail orientations might have cost D4 to lose significant polar contacts with Mpro pocket residues mediated by the ligand's terminal polar functionalities (guanidine, carboxylic group, and central amide) causing a compromised ligand-target pocket accommodation.

Thus, the depicted differential conformational changes among the onnamide family members highlight the impact of the nature of the aliphatic tail substitution for guiding preferential ligand–target stability. Regarding the reference ligands, both O6K and N3 are comparable proteomimetic ligands with several rotatable bonds exhibiting significant rotation at their dihedral/torsion angles. Interestingly, such inherited flexibility caused both ligands to twist along an anti-clockwise direction yet being maintained within the same orientations in respect to the Mpro pocket site (Fig. 5F and G). Notably, slightly higher orientation changes were illustrated for N3 over those of O6K providing reasonable explanations for the N3-complex RMSD trajectory fluctuations near the end of the MD simulation run.

**Fig. 6:**
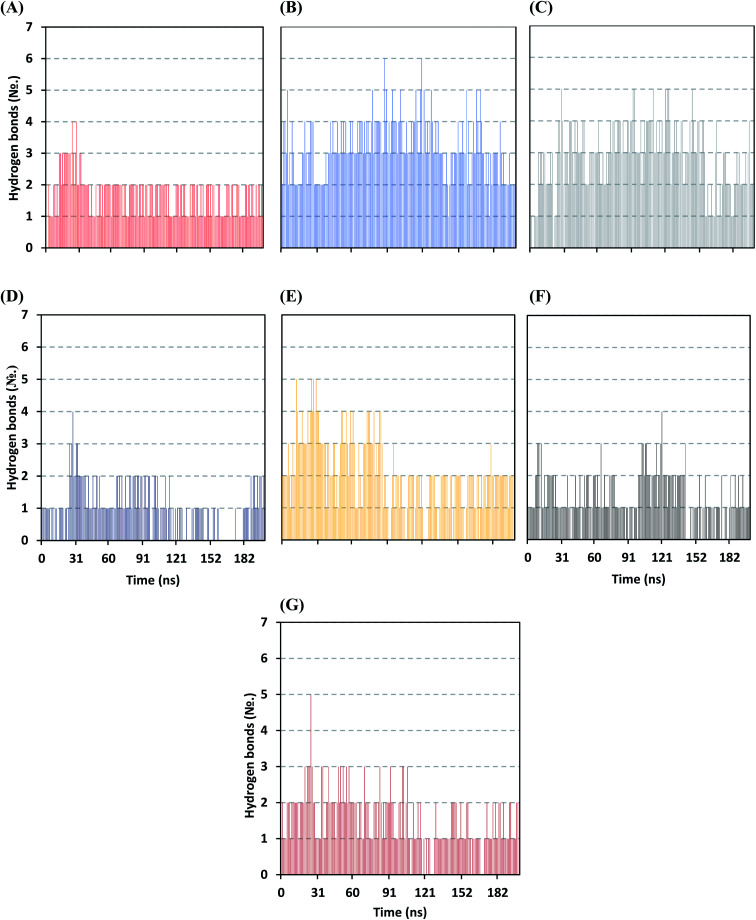
Time evolution of hydrogen bond number established between the ligand and Mpro protein at each simulated model. (A) D1; (B) D2; (C) D3; (D) D4; (E) D5; (F) O6K; (G) N3.

Further examining the proposed ligand–Mpro pocket stability was proceeded through monitoring the hydrogen bonding established between the ligand and target protein across the MD simulation trajectories. Plotting the number of formed hydrogen bonds between the simulated ligand and protein across the MD trajectories has revealed differential hydrogen bonding patterns across different systems. The simulated mycalamide family member, D1, showed a lower number of average hydrogen bond interactions as compared to members of the onnamide and theopederin family members. Lacking the terminal aliphatic tail causes D1 to possess the lowest number of hydrogen bond donors/acceptors available for predicted polar contacts with Mpro pocket residues. Additionally, D1 exhibited a nearly consistent number of hydrogen bonds with the target protein at the middle and till the end of the MD simulation time (Fig. 6A). This was highly correlated with the limited D1 conformational changes, particularly following the 100 ns MD simulation time frame, where the ligand exhibited comparable spatial orientation and pocket confinement.

On the contrarily, both the D2 and D3 of the onnamide family showed the highest number of hydrogen bond interactions across the whole MD simulation time frame (Fig. 6B and C). Showing such high polar interaction profiles could be reasoned since both D2 and D3 possess a higher number of hydrogen bond donors/acceptors (incorporated within both the core tetrahydropyran rings and tail substitution) besides showing significant ligand-pocket accommodation along with the whole simulation. As expected, the polar interaction profile of the above onnamide family members was not relevant for D4 since the latter exhibited poor pocket accommodation abounding the Mpro binding site within the middle of the MD simulation run. Interestingly, the D4-Mpro system lack any significant hydrogen bonding across several MD simulation frames, particularly around 130 and 160 ns simulation times (Fig. 6D). Regarding the simulated theopederin member D5-Mpro model, higher polar interacting profiles up to 5 hydrogen bonds were illustrated across the first 100 ns MD simulation trajectories (Fig. 6E). Afterward, a consistent number of hydrogen bonding was achieved till the end of the MD simulation except only for few frames missing relevant hydrogen bonding. Moving towards the two reference ligands, both O6K and N3 showed somewhat comparable hydrogen bonding profiles across MD simulation runs (Fig. 6F and G). This may be related to the close proteomimetic nature of both ligands incorporating similar N-terminal amino acids within their respective structures. However, N3 showed slightly higher average hydrogen bond numbers as compared to O6K owing to N3's longer residue sequence reflecting more hydrogen bond donors/acceptors incorporated within its structure.

#### 3.2.4. Binding-free energy calculations

In an attempt to further understand the nature of the ligand–protein interaction, explore the comparative ligand-binding site affinity, and obtain more information concerning individual ligand/residue contributions, the calculation of the binding-free energy was performed.^107^ In this regard, the MM/PBSA calculation was implemented for binding-free energy estimation, where higher negative binding energy explains more ligand affinity towards its respective target pocket.^91^ The MM/PBSA is considered of comparable accuracy to the free-energy perturbation approaches, yet with much smaller computational expenses.^91^ Using the solvent-accessible surface area (SASA), the only model of the free-binding energy calculation (Δ*G*_total_ = Δ*G*_molecular mechanics_ + Δ*G*_polar_ + Δ*GA*_polar_), as well as the single-trajectory approach, each energy term and their average values, were calculated across the representative frames extracted/saved from the whole 200 ns MD simulation trajectories. The single-trajectory approach was chosen since dealing with one trajectory of the ligand–Mpro complex rather than separated trajectories of the complex, receptor, and ligand was shown to be not only much faster but also less noisy.^108^ Adopting the calculation across the 200 ns MD simulation time course was rationalized by the rapidly attained equilibration/convergence of the complex RMSD trajectories for all investigated compounds and reference ligands following few initial MD frames (Fig. 2B).

To our delight, several investigated natural compounds depicted significant free-binding and affinity to the target's pocket (Table 5). The free binding energies of both the theopederin and onnamide family members (D2, D3, and D5) were estimated at significant negative values reaching up to 2-fold those of the two reference ligands. Considering the reported superior inhibition activity of the N3 ligand against SARS-CoV-2 Mpro,^105^ the obtained data for these latter drug class members highlights their potential activity against the same target enzyme. On the other hand, the less stabilized onnamide ligand, D4, depicted calculated binding-free energy being comparable to that of the reference ligands especially in relation to the N3-Mpro system. Finally, the calculated free binding energy for the D1-Mpro model was the least of all investigated ligands the thing that highlights the significant role of the extended aliphatic tail substitution for guiding the ligand–target interaction.

**Table 5:** Total binding-free energies and individual energy term (Δ*G*_total binding_ ± SD) concerning the promising isolated compounds and reference ligands at Mpro protein binding sites

Energy (kJ mol^−1^ ± SD)	Ligand–Mpro complex						
	D1	D2	D3	D4	D5	O6K	N3
Δ*G*_vanderWaals_	−131.24 ± 25.492	−174.99 ± 59.10	−222.49 ± 24.77	−121.81 ± 48.30	−138.44 ± 63.93	−163.04 ± 33.41	−149.43 ± 37.56
Δ*G*_Electrostatic_	−18.13 ± 10.47	−333.78 ± 44.68	−296.72 ± 87.32	−15.03 ± 12.01	−308.80 ± 138.05	−21.50 ± 16.40	−50.95 ± 22.43
Δ*G*_solvation;polar_	107.15 ± 31.30	362.00 ± 49.11	365.84 ± 90.83	67.39 ± 78.41	336.10 ± 79.19	125.32 ± 55.89	136.91 ± 69.41
Δ*G*_solvation;SASA_	−16.41 ± 2.42	−23.43 ± 3.88	−28.42 ± 1.90	−16.58 ± 6.19	−20.51 ± 7.59	−19.64 ± 3.57	−19.15 ± 4.09
Δ*G*_total binding_	−58.63 ± 15.60	−170.20 ± 30.57	−181.80 ± 21.42	−86.04 ± 86.01	−131.65 ± 115.50	−78.85 ± 15.49	−82.62 ± 25.48

Dissecting the obtained binding-free energy into its contributing energy terms showed a dominant energy contribution of the van der Waals interactions within the free-binding energy calculation of both the reference ligands. The higher hydrophobic and lower electrostatic energy contributions were assigned for O6K over N3 owing to the higher aromaticity and lower number of hydrogen bond donors/acceptors incorporated within the O6K core skeleton. The same preferential free binding energy contribution of the van der Waals potentials was depicted with D1-Mpro system where the absence of polar functionality related to the tail substitution deprived the ligand of relevant anchoring potentiality with the pocket polar residues. The above hydrophobic/electrostatic contribution profile was contrarily for the top-ranked isolated compounds (D2, D3, and D5) where the Coulomb's electrostatic potential energy was much higher than that of the van der Waals non-bonding interactions reaching up to ∼2-fold for D2 and D5 systems. The latter energy contribution pattern could be related to the highly polar extended tail substitutions, where these tails allowed D2, D3, and D5 to exhibit extended orientations within the Mpro pocket positioning their hydrophilically decorated bis-tetrahydropyran rings at one side of the pocket and the polar functionalities related to their long tail at the far pocket side (Fig. 5). Such depicted ligand–Mpro orientations have allowed these extended polyketides (D2, D3, and D5) to exhibit extensive polar interactions with Mpro pocket hydrophilic residues, whereas, D1 was deprived from such relevant anchoring potentiality for lacking the tail substitution. This came in good reason since the latter ligands showed higher number and frequency of hydrogen bonding with the Mpro protein pocket as compared to those of D1, N3 or O6K (Fig. 6). It is worth mentioning that the reported data within the current literature has considered the Mpro pocket to be more hydrophobic being deep, less solvent exposed, and with conserved hydrophobic pocket lining residues.^63,105,106^ Nevertheless, the ability of D2, D3, and D5 to establish favored strong polar interactions with the pocket's key residues allow them to be deeply anchored and attaining significant pocket specificity. This was obvious through the previously described conformational and hydrogen bonding analysis.

All above data confirms the significant role of the polar functionalities related to the terminal tail substitution for ligand anchoring within Mpro binding site. Notably, the presence of polar functionalities related to the terminal tail substitution could act as a double-bladed influencer on ligand–protein binding since such functional groups impose higher Δ*G*_solvation_ that might compromise the ligand anchoring since the binding process is a solvent-substitution approach. Thus, optimizing these isolated compounds through the introduction of ionizable groups yet with higher hydrophobic characteristics (*i.e.*, tetrazole functionality) would be suggested relevant for minimizing the Δ*G*_solvation_, extending the ligand–Mpro binding, and furnishing potential target inhibition. Finally, the total non-polar interactions (Δ*G*_vanderWaals_ plus Δ*G*_SASA_) were higher for the top-binding compounds (D2 and D3) as compared to those of reference ligand N3 the thing that would have been directly related to the pocket's large surface area. Being hydrophobic and with a large surface area, the Mpro binding site would favor higher non-polar interactions with D2 and D3 since the latter ligands are capable to attain a more extended conformation within the target's pocket.

**Fig. 7:**
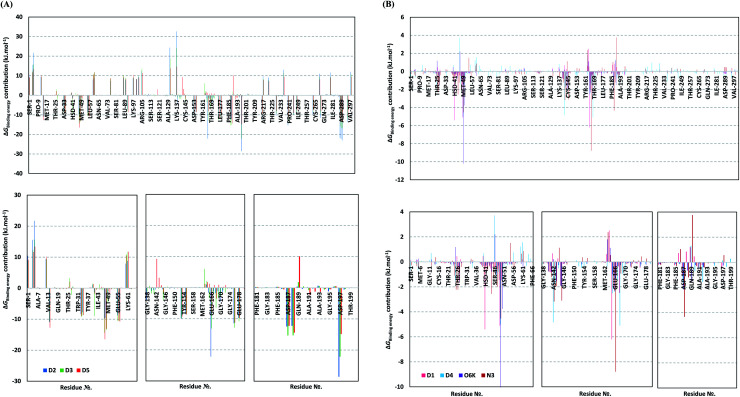
Binding-free energy/residue decomposition illustrating the protein residue contribution at ligand–protein complex Δ*G*_total binding_ calculation. (A) Top-binding ligands; D2, D3, and D5. (B) Lower-binding ligands; D1, D4; as well as reference compounds; O6K and N3. Lower panels are expanded versions of three designated residue regions (Ser1-Phe66, Gly138-Asn180, and Phe181-Ile200) of the upper panel.

For gaining more insights regarding ligand–residues interactions, the binding-free energy decomposition within the *g_mmpbsa* module was utilized to identify the key residues involved within the obtained binding free energies.^91^ Interestingly, similar residue-wise energy contribution patterns were assigned for the top-binding onnamide and theopederin family members, D2, D3, and D5 (Fig. 7A). This was of no surprise since these ligands depicted comparable energy terms as well as total binding-free energy values. On the other hand, the family member compound (D1) shares, to some extent, several residue-wise energy contribution patterns similar to those of the three top-binding ligands, yet of lower magnitude (Fig. 7B). As expected, the D4 compound showed the lowest residue-wise energy contributions with most of the residues involved with the top-binding compounds. Additionally, several Mpro pocket-specific residues showed positive energy contributions with D4 inferring repulsion effect and unfavoured ligand-pocket accommodation.

Notably, residues of the S2 subsites and their vicinal residues showed the most extended and one of the highest residue-binding energy contributions with values up to two-digit kJ mol^−1^ values. Residues such as; Glu47, Asp48, Met49, Glu55, Asp56, Asp187, and Arg188 illustrated very high energy contributions ranging from −6.20 ± 0.761 kJ mol^−1^ and up to −15.32 ± 0.98 kJ mol^−1^. Similar energy contribution patterns, yet with smaller magnitudes, were assigned for the S3 subsite residues and vicinal amino acids including Leu167, Pro168, Thr169, Asp176, and Glu178 (−5.10 ± 1.63 up to −11.46 ± 0.38 kJ mol^−1^). The profound and widespread energy contribution of the S2 residues came in great concordance with the previously described RMSF analysis inferring the crucial role of these residues within the ligand anchoring. Contributions of the key S1 sub-pocket residues were only assigned high for Glu166 exhibiting high residue-associated energy contribution of −22.173 ± 2.26 kJ mol^−1^ and −13.16 ± 4.21 kJ mol^−1^ for D2 and D3, respectively. On the contrary, the rest of the S1 residues either showed low negative (Phe140, Leu141, Asn142) or even positive energy contribution values as with His163 inferring their limited or unfavoured role for ligand binding, respectively.

Insignificant energy contributions were depicted for the residues of the S1′ sub-pocket, however, only a single contribution for the catalytic dyad, His41 (−1.37 ± 1.25 to −8.99 ± 1.60 kJ mol^−1^) was worth finding except for D4 (−0.29 ± 1.52 kJ mol^−1^). It is worth mentioning that the three top-binding ligands, D2, D3, and D4 showed a distinct high positive residue-wise contribution with several ionizable residues surrounding the pocket residues and their most vicinal ones. These residues include Arg4, Lys5, Arg60, Lys88, Lys90, Lys97, Lys100, Lys102, Arg105, Arg131, and Lys137, being cationic and showing high positive energy contribution up to ∼33 kJ mol^−1^. Such findings suggest the role of these residues for imposing repulsive effects favoring the confinement of D2, D3, and D5 within the Mpro binding site. Finally, both references showed significant contributions only for S1 (Leu141, Asn142, Glu166), S2 (Met49, Asp187, Arg188) without relevant contributions for S3 residues (Fig. 7B). Unlike the above-investigated compounds, higher energy contribution of the S1′ catalytic Cys145 (−1.93 ± 1.23 and −3.10 ± 3.59 kJ mol^−1^) over that of other dyad His41 (−0.31 ± 0.40 and −0.53 ± 0.71 kJ mol^−1^) were depicted for O6K and N3, respectively.

### 3.3. Structure–activity relationship study

**Fig. 8:**
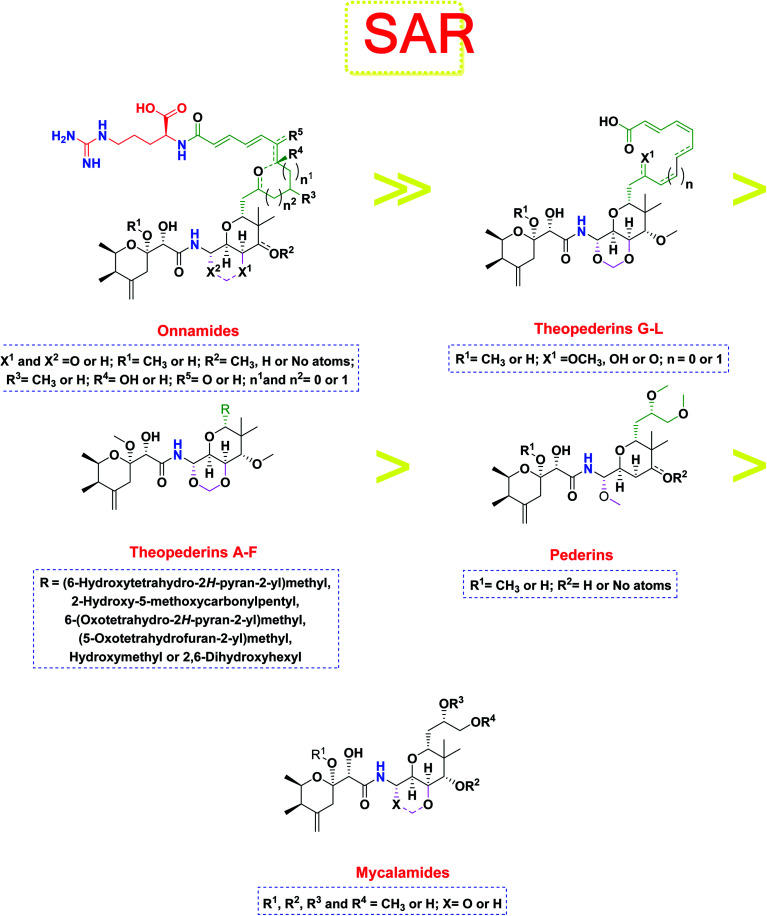
General structure–activity relationship study of the tested structurally related pederin marine compounds.

The investigated compounds share a common core composed of two tetrahydropyran rings; one of them possesses an aminal moiety whilst the other bears an acyl functional group, which are coupled *via* an amide linkage. Despite possessing a similar core, the estimated highly variable binding score might be a reflection of the presence or absence of some moieties, additional rings, functional groups, and/or variance of the oxygenation pattern (numbers and positions of mainly hydroxyl and/or methoxy groups). Relating the structures (1–34; Schemes 1–4) to the estimated binding scores (Table 1) suggests that the most influential structural element on the predicted binding score is the carbon chain present as a substituent on the tetrahydropyran ring bearing the aminal moiety, especially when contains a terminal arginine residue (Fig. 8). The impact of other structural elements was relatively lower on the calculated binding sore.

Thus, the high scores of onnamides (9–19 and 21) that surpassed the calculated binding score of the co-crystallized inhibitor (O6K, 35) of the dimeric form (Table 1) might be attributed mainly to the presence of 10–12 unsaturated acyl carbon chain (green-colored; Fig. 8) coupled with an arginine residue (red-colored; Fig. 8). This might be confirmed upon comparison with the binding score onnamide (20) that lacks the arginine residue, which possessed a lower binding score relative to the calculated score of the co-crystallized inhibitor (35) of the dimeric form. Probably, the guanidinic moiety of the arginine reside offers favorable binding interactions. Amongst onnamides, the best score was for 6,7-dihydro-onnamide A (11) whose substituent acyl chain coupled with arginine is of 12 carbons length and incorporates *trans*-8,10-diene. Its calculated binding score (−10.19 kcal mol^−1^) was much better than the calculated score of the co-crystallized inhibitor (35) of the dimeric form and almost similar to the calculated score of the co-crystallized inhibitor (36) of the monomeric form (Table 1). Such a high score of 6,7-dihydro-onnamide A (11) might be a result of an optimum distance between arginine residue and the core tetrahydropyran rings offered by the 12 carbons-length *trans*-2,4-diene substituent. This might be inferred from the found relatively lower score of compounds possessing 10 carbons-length *trans*-2,4-diene substituents (12 and 13) in comparison with compounds possessing 12 carbons-length substituents. Although of minimal effect, it was found that compounds having 17-hydroxy functionality (12) are slightly better than 17-oxo functionality (13). However, this lowering effect for conversion of the corresponding 19-hydroxy functionality in 6,7-dihydro-onnamide A (11) to a 19-oxo functionality in 6,7-dihydro-oxo-onnamide A (18) was much larger. Nevertheless, this order was reversed as the conversion of the corresponding 19-hydroxy functionality in onnamide D (15) to a 19-oxo functionality in oxo-onnamide A (19) resulted in minimal enhancement of the binding score. Introduction of a double bond at 6-position of the 12 carbon-length *trans*-2,4-diene to become *trans*-2,4,6-triene (pseudo-onnamide A (17), onnamide E (16), onnamide A (9), oxo-onnamide A (19), and onnamide D (15)) afforded notably high scoring compounds, but less than 6,7-dihydro-onnamide A (11). Similarly, the formation of a cyclic hemiacetal between the 19-hydroxy functionality and an introduced 15-carbonyl functionality in side-chain affording an additional tetrahydropyran ring (onnamide C (14)) resulted in a notably high scoring compound but, also, less than 6,7-dihydro-onnamide A (11). The scores of these compounds were very close but in order pseudo-onnamide A (17) > onnamide C (14) > onnamide E (16) > onnamide A (9) > oxo-onnamide A (19) > and 6,7-dihydro-oxo-onnamide A (18) ≈ onnamide D (15). As revealed from these results, the 1,3-dioxane ring fused to tetrahydropyran ring bearing the aminal moiety in onnamides has little effect as onnamide D (15) and onnamide E (16).

As mentioned above, the second most influential structural element was the side chain. This was very clear in theopederins family whose members bear a 10 or 12 carbons-length chain, a 6-carbons-length chain, or the chain might be almost truncated. In general, members having 10 or 12 carbons-length chains possessed a relatively higher calculated binding score (theopederins G–L; Fig. 8). The best score amongst this family was for theopederin G (29; −8.45 kcal mol^−1^) which incorporates a 10 carbons-length *trans*-2,4-diene chain which is very close to the score of theopederin I (31; −8.43 kcal mol^−1^) which incorporates a 12 carbons-length *trans*-2,4,6-triene chain. Meanwhile, theopederin J (32) incorporating a 12 carbons-length *trans*-2,4-diene chain showed a lower binding score. In comparison, compounds theopederin K (33) and theopederin L (34) having *trans*-2,4,7-triene chain showed relatively lower scores. As noted above, conversion of the hydroxy functionality in the chain of theopederin G (29) to an oxo functionality in theopederin H (30) results in a slight lowering of the binding score. In addition, conversion of the hydroxy functionality in the chain of theopederin L (34) into a methoxy functionality in theopederin K (33) results in a slight lowering of the binding score, too. It might be inferred that the hydrogen of this hydroxyl group might be involved in some interaction. Among compounds having 6 carbons-length chains, only those possessing 5-membered lactone or tetrahydropyran rings showed binding scores better than −8.0 kcal mol^−1^ (theopederins D (26) and A (22 and 23 for α- and β-epimers respectively)). Thus, compounds whose 6 carbons-length chains incorporated 6-membered lactone ring (theopederins C (25)), non-cyclic ester (theopederins B (24)), or diol (theopederins F (28)) showed −7.99–7.89 kcal mol^−1^ binding scores. The −7.12 kcal mol^−1^ binding scores of theopederins E (27) whose chain is almost truncated, which is a much less favorable binding score might bolster the inferred importance of this chain for eliciting good binding scores. Considering this family, the binding scores were in order theopederin G (29) ≈ theopederin I (31) > theopederins D (26) > α-epimer of theopederins A (22) > β-epimer of theopederins A (23) > theopederin J (32) ≈ theopederin H (30) > theopederin L (34) > theopederins F (28) > theopederins C (25) > theopederin K (33) > theopederins B (24) > theopederins E (27).

In the case of pederins, the size of the influential side chain is 3 carbons-length. Such chain bears two methoxy groups which are among the features that distinguish pederins from mycalamides in which one of/both of these methoxy groups exist(s) as hydroxyl group(s). As noted above, the 1,3-dioxane ring fused to tetrahydropyran ring bearing the aminal moiety has little effect. Accordingly, pederins showed good binding scores although they lack such 1,3-dioxane ring. The highest score among pederins was triggered by pederin (1) which has a derivative 2-methoxytetrahydropyran as the core tetrahydropyran ring bearing the acyl functional group. Conversion of the 4-hydroxytetrahydropyran that bears the aminal moiety in pederin (1) into tetrahydro-4-pyrone in pederone (3) results in the minimal reduction of the calculated binding score. However, the noticed reduction of the calculated binding score upon replacement of the derivative of 2-methoxytetrahydropyran in pederin (1) by the corresponding derivative of 2-hydroxytetrahydropyran in pseudopederin (2) suggests a potential role for this methoxy function.

This might be supported by the found much lower binding score of mycalamide E (6) that contains a similar 2-hydroxytetrahydropyran moiety relative to other mycalamides possessing 2-methoxytetrahydropyran. Considering the effects of the other structural elements, mycalamides in which the presence of 2-methoxytetrahydropyran ring was combined with a side chain bearing two hydroxy groups; mycalamide A (4) and mycalamide D (8), were the best scoring mycalamides. Out of them, mycalamide A (4) was better, probably because the other 4-hydroxy functionality on the other tetrahydropyran ring of mycalamide D (8) is converted in a 4-methoxy group. When the side chain possessed one methoxy and one hydroxyl group; mycalamide B (5) and mycalamide C (7), there was a binding score reduction relative to the corresponding compounds. The more score reduction was associated with mycalamide C (7) which lacks also the 1,3-dioxane ring fused to tetrahydropyran ring bearing the aminal moiety. Over members of pederins and mycalamides, the best scores order was pederin (1) > mycalamide A (4) > pederone (3) > mycalamide D (8) > mycalamide B (5) > mycalamide C (7) > pseudopederin (2) > mycalamide E (6).

## 4. Conclusion

Thirty-three focused marine natural products related to the pederins, mycalamides, onnamides and theopederins polyketide families were comprehensively examined for their binding affinities against the dimeric form of the Mpro of SARS-CoV-2 using an integrated set of computational methods including molecular docking and molecular dynamics simulations studies. Our results disclosed that most of the examined members are exhibiting promising binding scores and modes especially dihydro-onnamide A (11), onnamide C (14), and pseudo-onnamide A (17) which exceeded the co-crystallized inhibitor (O6K, 34) binding score. Molecular dynamic simulation illustrated the preferential stability of almost all investigated compounds at the Mpro binding site, however, D2, D3, and D5 exhibited the most preferential stability and highest free binding energies being nearly 2-fold those of the potent Mpro inhibitors, O6K and N3. For future lead development and optimization of these top stable ligands, it is recommended that introducing ionizable groups yet with higher hydrophobic characteristics (*i.e.* tetrazole functionality) would be relevant for minimizing the Δ*G*_solvation_, extending the ligand–protein binding, as well as furnishing potential target inhibition. Furthermore, an interesting SAR study was performed to correlate the diverse structural modifications on the proposed activity. Those encouraging findings highlight that such fantastic molecular architectures could open a gate for the development of promising antiviral treatments for beating the COVID-19 outbreak. Moreover, considering flexible chemical syntheses for plenty of those compounds/congeners or structurally related modified members could be urged for more preclinical and clinical examinations either alone or in combination with each other for COVID-19 management.

## Author contributions

Conceptualization: Amr El-Demerdash, Ahmed A. Al-Karmalawy, Tarek Mohamed Abdel-Aziz, and Ahmed H. E. Hassan. Methodology: Ahmed A. Al-Karmalawy, and Khaled M. Darwish. Validation: Ahmed A. Al-Karmalawy, and Khaled M. Darwish. Formal analysis: Ahmed A. Al-Karmalawy, and Khaled M. Darwish. Investigation: Amr El-Demerdash, Ahmed A. Al-Karmalawy, Tarek Mohamed Abdel-Aziz, Khaled M. Darwish, and Ahmed H. E. Hassan. Resources: Amr El-Demerdash, Ahmed A. Al-Karmalawy, Tarek Mohamed Abdel-Aziz, Khaled M. Darwish, and Ahmed H.E. Hassan. Data curation: Ahmed A. Al-Karmalawy, Khaled M. Darwish, and Ahmed H. E. Hassan. Writing original draft: Amr El-Demerdash, Ahmed A. Al-Karmalawy, Tarek Mohamed Abdel-Aziz, Sameh S. Elhady, Khaled M. Darwish, and Ahmed H. E. Hassan. Writing-review & editing: Amr El-Demerdash, Ahmed A. Al-Karmalawy, Tarek Mohamed Abdel-Aziz, Khaled M. Darwish, and Ahmed H. E. Hassan.

## Conflicts of interest

There are no conflicts to declare.

## Supplementary Material

RA-011-D1RA05817G-s001
