# Supplementary material for: Investigating the structure–activity relationship of marine natural polyketides as promising SARS-CoV-2 main protease inhibitors
Source: RSC Adv. 2021 Sep 22;11(50):31339–63. doi: 10.1039/d1ra05817g (PMC9041294; doi:10.1039/d1ra05817g)
Supplement: RA-011-D1RA05817G-s001 [file RA-011-D1RA05817G-s001.pdf]

Electronic Supplementary Material (ESI) for RSC Advances.
This journal is © The Royal Society of Chemistry 2021

Investigating the Structure-Activity Relationship of Marine Natural Polyketides as Promising SARS-CoV-2 Main Protease Inhibitors

Amr El-Demerdash[1,2,⊥,*], Ahmed A. Al-Karmalawy [3,⊥,*], Tarek Mohamed Abdel-Aziz[4,5], Sameh S. Elhady[6], Khaled M. Darwish[7], and Ahmed H.E. Hassan[8,*]

[1] Organic Chemistry Division, Chemistry Department, Faculty of Science, Mansoura University, Mansoura 35516, Egypt.
[2] Metabolic Biology & Biological Chemistry Department, John Innes Centre, Norwich Research Park, Norwich NR4 7UH, UK.
[3]Department of Pharmaceutical Medicinal Chemistry, Faculty of Pharmacy, Horus University-Egypt, New Damietta 34518, Egypt.
[4] Department of Cellular and Integrative Physiology, University of Texas Health Science Centre at San Antonio, San Antonio, Texas 78229-3900, USA.
[5] Zoology Department, Faculty of Science, Minia University, El-Minia 61519, Egypt.
[6]Department of Natural Products, Faculty of Pharmacy, King Abdulaziz University, Jeddah, Saudi Arabia.
[7] Department of Medicinal Chemistry, Faculty of Pharmacy, Suez Canal University, Ismailia, Egypt.
[8]Department of Medicinal Chemistry, Faculty of Pharmacy, Mansoura University, Mansoura 35516, Egypt.

Figure S11. Side-view of the full ligand-Mpro complexes embedded within the TIP3P system. (**A**) D1; (**B**) D2; (**C**) D3; (**D**) D4; (**E**) D5; (**F**) O6K; (**G**) N3. Proteins are represented as cartoon coloured green, ligand atoms are displayed as yellow spheres, while as TIP3P water molecules as lines. The ions are hidden for clarity.

(A) **(B)**

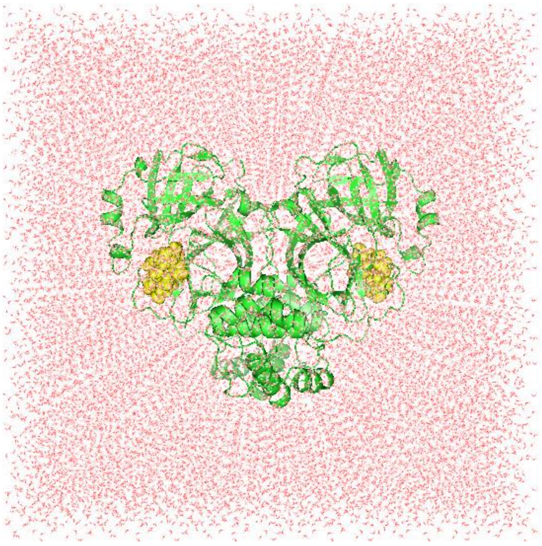

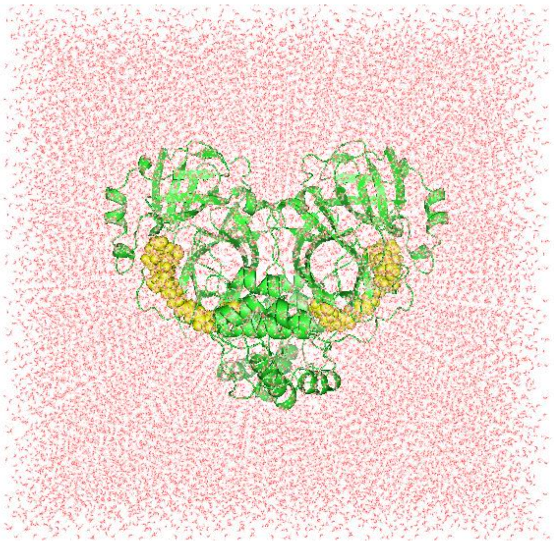

(C)

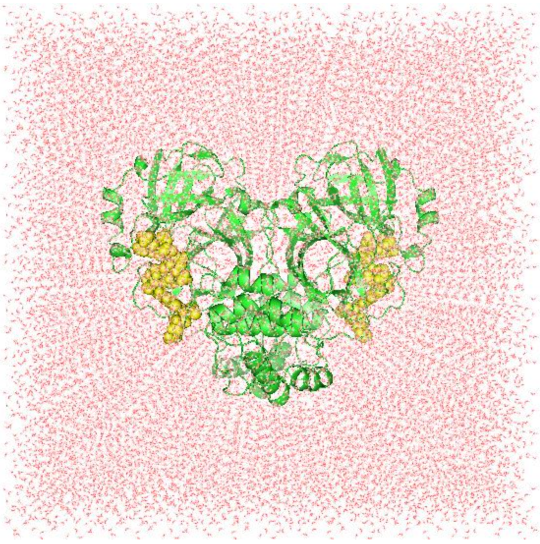

(D)

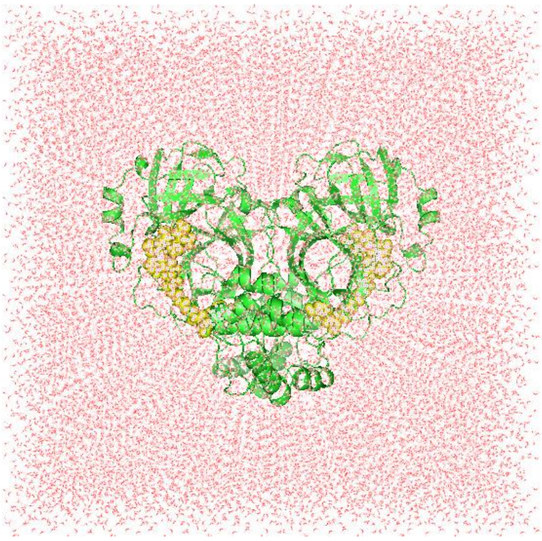

(E)

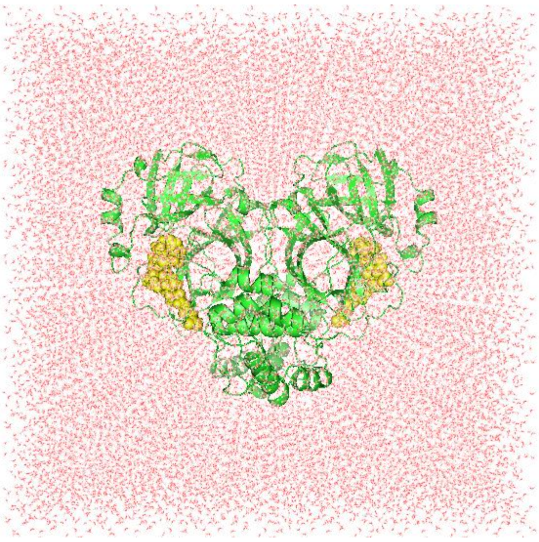

(F)

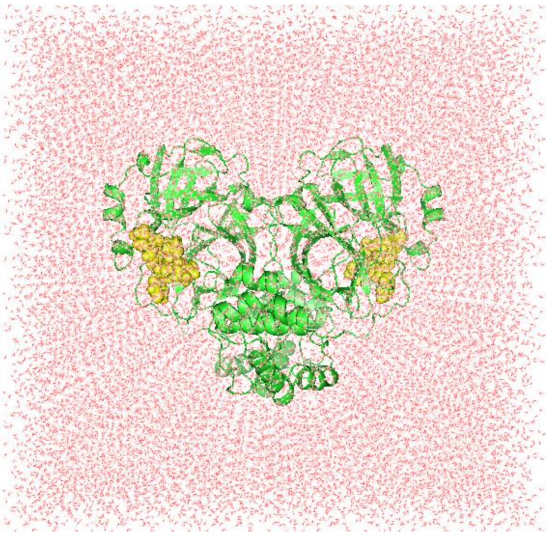

(G)

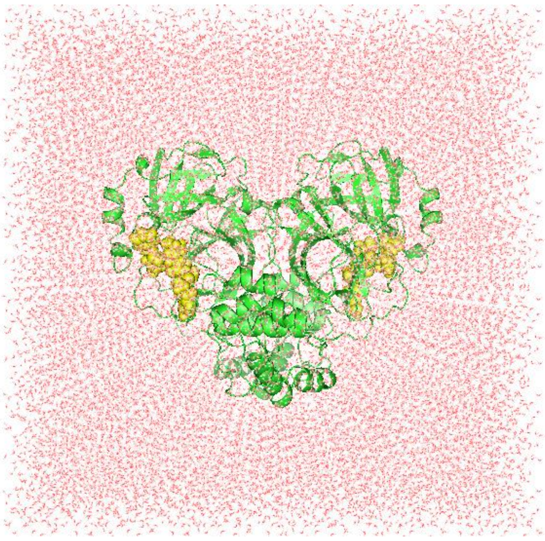

Figure SI2. 2D binding and positioning of the five examined marine products (**1**, **11**, **14**, **17**, and **29**) besides the docked co-crystallized inhibitors (**35** and **36**) towards the binding pocket of SARS-CoV-2 Mpro.

**Compound**	**2D pocket binding (SARS-CoV-2 Mpro)**
Pederin (**1**)	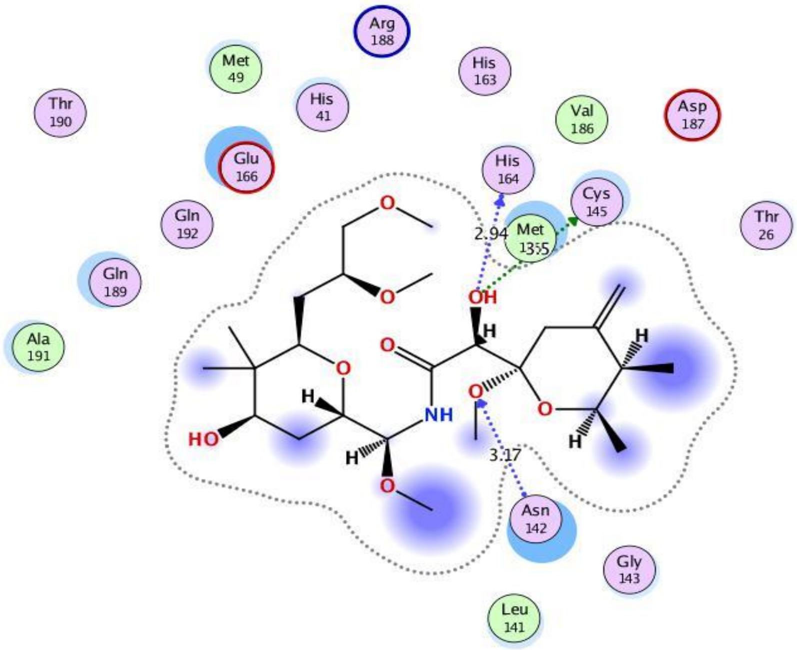
Dihydro-onnamide A (**11**)	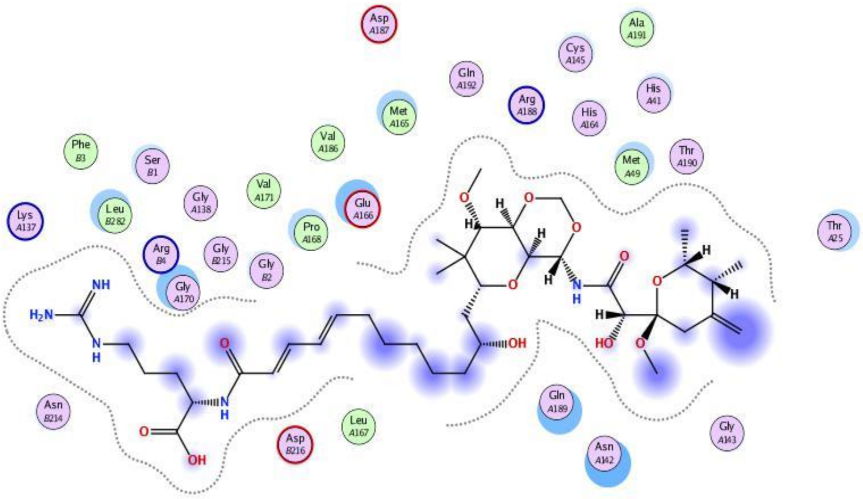

<table>
<tr><td>Onnamide C
(**14**)</td><td>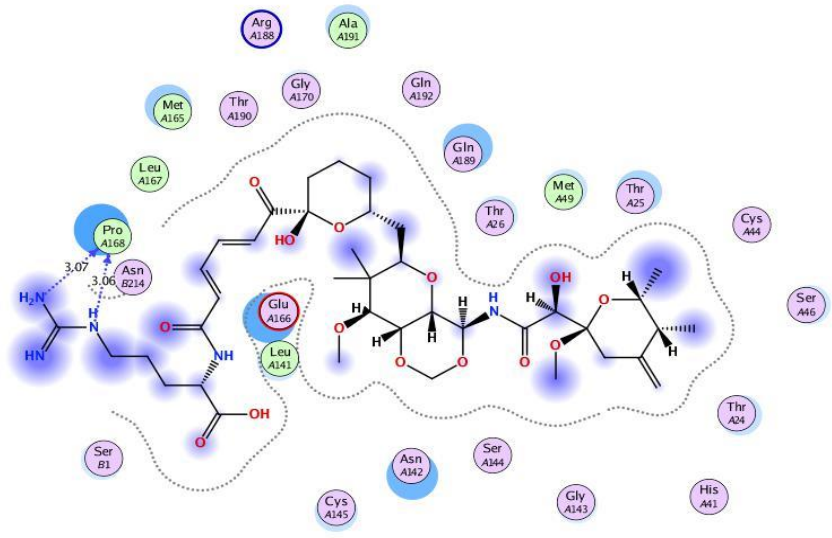
</td></tr>
<tr><td>Pseudo-onnamide A
(**17**)</td><td>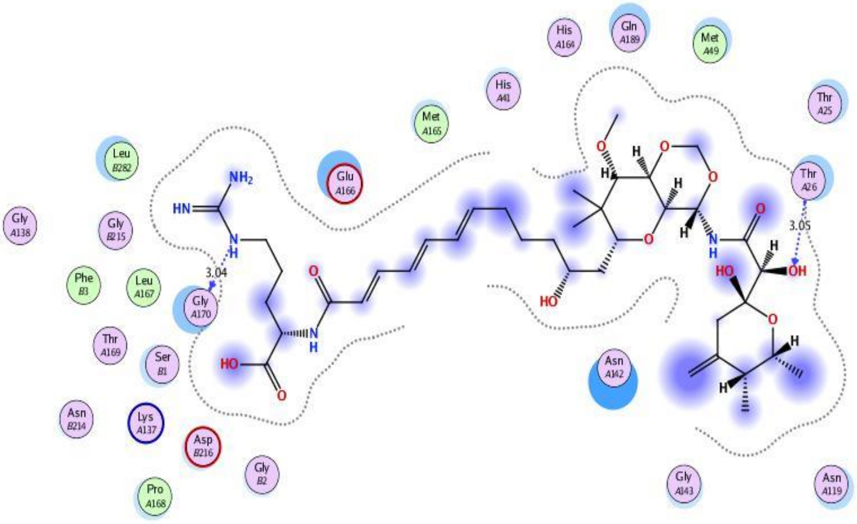
</td></tr>
<tr><td>Theopederin G
(**29**)</td><td>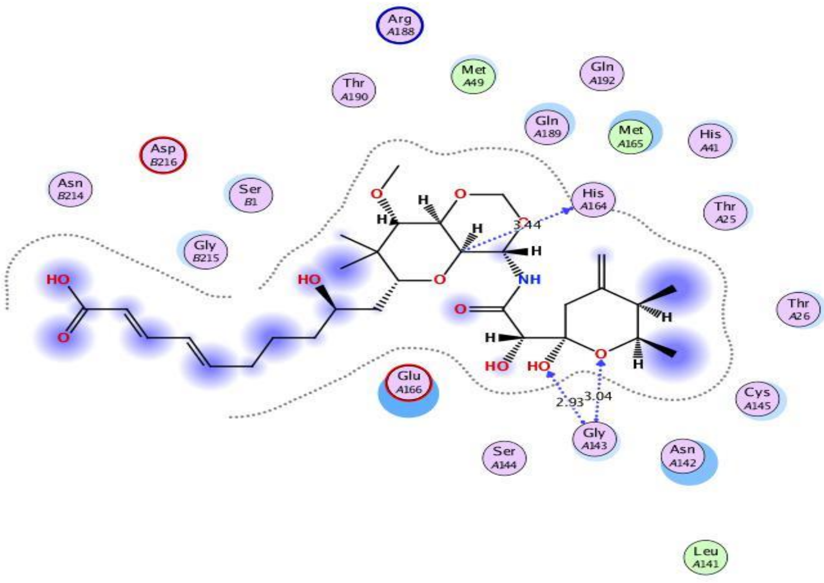
</td></tr>
</table>

<table>
<tr>
<td>6Y2G
co-crystallized inhibitor
(O6K, 35)</td>
<td>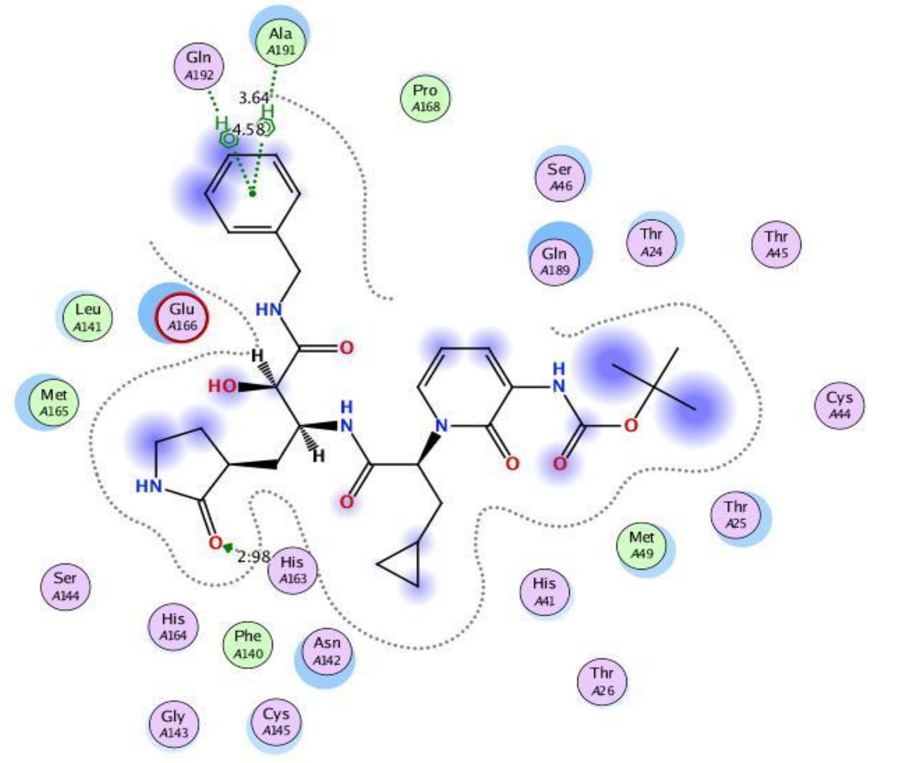
</td>
</tr>
<tr>
<td>6LU7
co-crystallized inhibitor
(N3, 36)</td>
<td>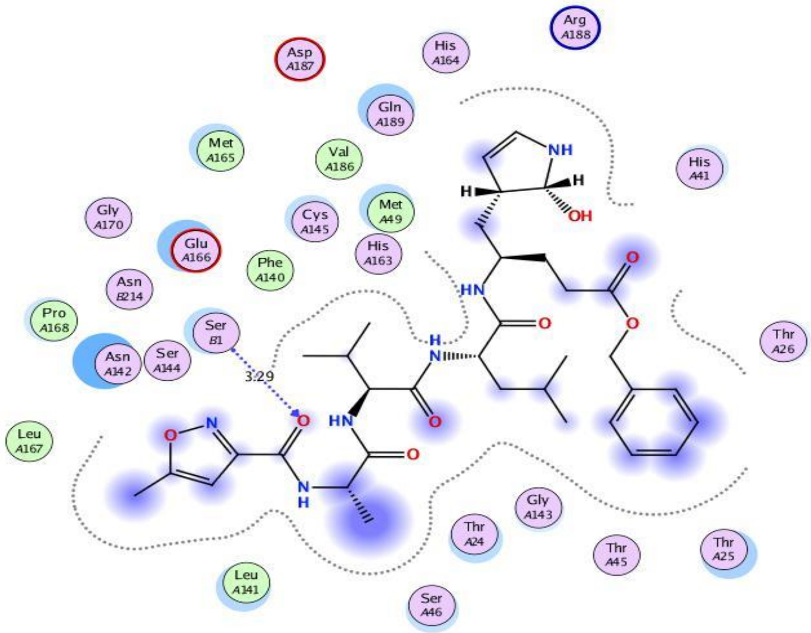
</td>
</tr>
</table>

35
